# Insight into the Influence of Ecological Factors on Shaping Distribution Patterns of *Camptotheca acuminata* for Conservation and Management

**DOI:** 10.3390/plants14101466

**Published:** 2025-05-14

**Authors:** Taojing Wang, Yuchen Li, Teja Manda, Jie Lin, Tianyu Huang, Yao Zhang, Liming Yang

**Affiliations:** State Key Laboratory of Tree Genetics and Breeding, College of Biology and the Environment, Nanjing Forestry University, Nanjing 210037, China; liyuchen@njfu.edu.cn (Y.L.); teja.manda27@gmail.com (T.M.); linjie200103@163.com (J.L.); huangtianyu2@126.com (T.H.); 202312082@njfu.edu.cn (Y.Z.); yangliming@njfu.edu.cn (L.Y.)

**Keywords:** *Camptotheca acuminata* Decne., MaxEnt, geographical distribution, environmental factor, climate change, ecological conservation

## Abstract

*Camptotheca acuminata* Decne. is an endemic and valuable tree species in China that is renowned for its medicinal and economic value due to secondary metabolites like camptothecin, a potent anti-cancer compound. With wild resources dwindling, it is a key protected species. Predicting and analyzing its suitable habitats under different future environmental scenarios is essential for conservation, introduction, development, and planting strategies. This study used 1008 distribution points and 32 environmental factors, applying the MaxEnt v3.4.4 model and ArcGIS v10.7 software to predict *C. acuminata*’s potential distribution under four greenhouse gas emission scenarios (RCP2.6, RCP4.5, RCP6.0, and RCP8.5) for the present, 2050, and 2070. This study identifies the key environmental factors influencing its distribution and analyzes habitat trends under various ecological scenarios. The dominant environmental factors are Bio6 (contribution 23%; importance 59.8%), human activity factor (contribution 18.6%; importance 15.7%), Slope2 (contribution 1%; importance 7%), Slope3 (contribution 5.1%; importance 3.4%), elevation (contribution 0.9%; importance 1.7%), and Bio14 (contribution 41.2%; importance 1%). The total potential suitable habitat area for *C. acuminata* is 1.5796 × 10^4^ km^2^. Except under RCP8.5, where the habitat area continuously increases, the habitat area shows a trend of first increasing and then decreasing. When human activity is considered, the total potential suitable habitat area is 1.8495 × 10^4^ km^2^, with a consistent decrease under all scenarios except RCP8.5. Centroid migration analysis shows that, driven by global warming, the suitable habitats for *C. acuminata* are shifting toward higher latitudes. This study provides theoretical support for the conservation, resource management, and germplasm protection of *C. acuminata* under future ecological and environmental changes.

## 1. Introduction

*Camptotheca acuminata* Decne., a plant of the *Nyssaceae* family and *Camptotheca* genus, is a perennial deciduous broadleaf tree native to subtropical regions. It is an endemic species in China, distributed in the middle and lower Yangtze River Basin and the southern areas of China [1]. *C. acuminata* is valuable in medicine, landscaping, materials, and environmental conservation. The alkaloid camptothecin, found in the roots, bark, and fruits of the tree, has demonstrated effectiveness in treating leukemia, cancer, diabetes, and cardiovascular diseases [2]. Wild resources of *C. acuminata* in China are rapidly depleting, making it one of the 120 extremely small population wild plants in the country and placing it on the verge of extinction [3], particularly due to the destructive effects of human activities, which have led to widespread deforestation and a sharp decline in wild populations. Given the significant economic, medicinal, ecological, and societal value of *C. acuminata*, studying its distribution characteristics, the ecological and climatic factors influencing its geographical distribution, and predicting its future distribution under different ecological scenarios is of great importance and value.

The geographical distribution of a species is a spatial characteristic shaped by ecological, environmental, and human activity factors, while its genetic structure, together with environmental conditions, determines all phenotypic traits of the species. The genetic structure can buffer environmental changes to a certain extent, but adaptations beyond specific thresholds rely on genetic evolution. Among these factors, climate is one of the most critical determinants influencing the phenotypic traits of all plants [4,5]. Climate change can affect the geographical distribution range of species, alter species composition and richness, and subsequently impact biodiversity as well as the structure and function of ecosystems [6,7,8]. The continuous rise in global temperatures each year and the expected ongoing climate warming will have profound and unimaginable effects on Earth’s biodiversity and ecosystems [9]. Therefore, analyzing the potential geographical distribution of species under different climate conditions is crucial for biodiversity conservation, ecological restoration, and the sustainable maintenance of ecosystems.

According to reports from the Intergovernmental Panel on Climate Change (IPCC), global temperatures are expected to rise annually, with the Earth’s average temperature projected to increase by 0.3 to 4.8 °C by 2100 compared to the period between 1986 and 2005 [9]. Climate change induced by human activities and other factors will lead to alterations in seasonal temperatures, including changes in precipitation, low temperatures, and extreme climate events, which will subsequently affect the geographical distribution suitable for plant growth. Previous studies have shown that global warming can promote the migration of plants to higher altitudes and latitudes [10]. Chen et al. used MaxEnt to find that future climate change will cause the potentially suitable habitat of *Paris polyphylla* in Yunnan to expand toward higher altitudes and latitudes. Under different scenarios, the highly suitable habitat areas show varying degrees of reduction [11]. Zhang et al., through the prediction and analysis of the geographical distribution of 2319 woody plant species in Yunnan, China, found that under the most extreme climate change scenario, the maximum extinction rate is approximately 6%. However, under this scenario, up to 1400 species are expected to lose more than 30% of their current range [12]. Conversely, changes in species distribution ranges will also impact regional climate change, as surface vegetation strongly influences atmospheric characteristics [13]. Under the current global climate warming context, predicting the spatial changes and migration trends of suitable distribution areas for endangered species threatened by climate change has become one of the latest research hotspots and main directions in conservation biology and forest ecology [14].

With the deepening study of species’ spatiotemporal distribution, Species Distribution Models (SDMs) have rapidly developed and are widely used to quantify the relationship between species habitat selection and complex environmental factors. Common SDMs include Generalized Linear Models (GLMs), Generalized Boosted Models (GBMs), Multivariate Adaptive Regression Splines (MARSs), Maximum Entropy Model (MaxEnt), Artificial Neural Networks (ANNs), Random Forest (RF), and Support Vector Machines (SVMs) [15]. Among these, the MaxEnt model, based on the maximum entropy theory and written in JAVA v8 is a modeling software that uses the jackknife method to assess the importance of environmental factors and quantitatively describes the impact of these factors on species' habitats. Since MaxEnt has low requirements for distribution data and provides stable and reliable predictions, it has been widely applied in species habitat suitability assessments and studies of species’ spatiotemporal distribution patterns [15,16,17,18].

Predicting the future suitable distribution areas of *C. acuminata* and analyzing the main environmental factors influencing its distribution is crucial for effective resource conservation, introduction, development, and planting zone planning. This study uses the MaxEnt model, along with *C. acuminata*’s geographical distribution data, to analyze its distribution characteristics under current climate conditions. It also examines the potential distribution and dynamic changes in *C. acuminata* in China in 2050 and 2070 under different carbon emission scenarios.

## 2. Results

### 2.1. Environmental Factor Selection Results Affecting the Distribution of C. acuminata

**Figure 1:**
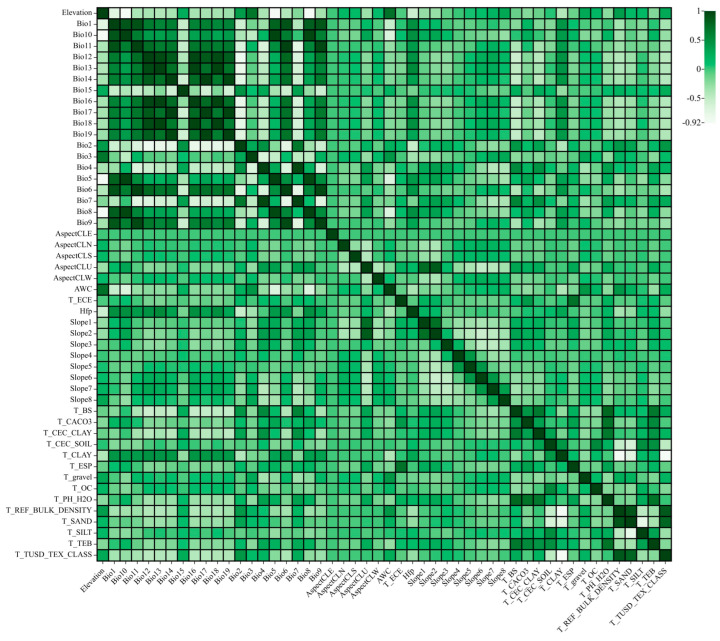
Correlation analysis of environmental factors.

Based on the contributions from the first simulation and the correlations between factors (Figure 1), 32 environmental factors were selected. These 32 factors were then re-simulated. By analyzing the contribution rates, permutation importance values, and jackknife method results from the MaxEnt output, the dominant environmental factors influencing the potential geographical distribution of *C. acuminata* in China were identified. The jackknife method was used to analyze the relationship between the distribution of suitable habitats for *C. acuminata* and different environmental factors, while the permutation importance value indicates the strength of the model’s dependence on each variable. According to the criteria, the blue striped area, representing “only this variable”, indicates that as the strip length increases, the predictive ability of the species distribution for that variable strengthens. The cyan striped area, representing “all except this variable”, indicates the sum of the remaining variables’ contribution to the training score. If the “all except this variable” score is low, it suggests that the variable contains unique information, which is also of significant importance for the species’ distribution. The cumulative contribution rate of all environmental variables in the constructed model is represented by the red-striped area.

**Figure 2:**
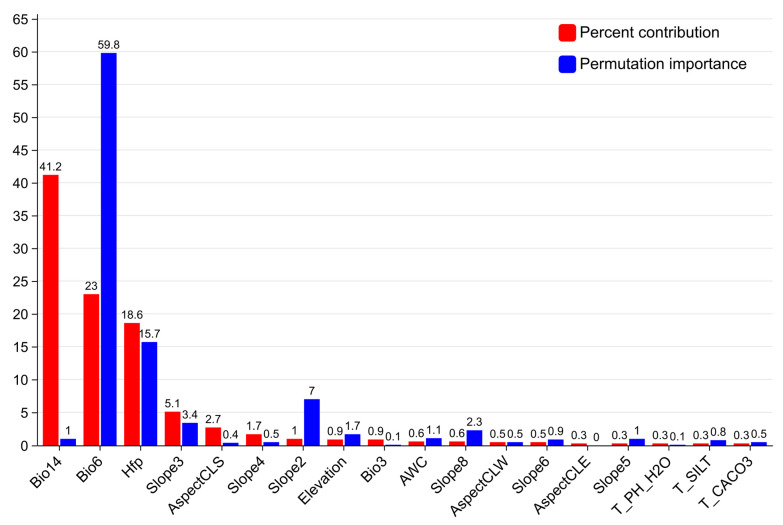
The red bar is the percent contribution of environmental factors; the blue bar is the permutation importance of environmental factors.

Based on the percent contribution (Figure 2), the environmental factors with contributions exceeding 5% are Bio14 (41.2%), Bio6 (23%), Hfp (18.6%), and Slope3 (5.1%). The cumulative contribution rate of these four factors reaches 87.9%, making them the dominant variables influencing the distribution of C. acuminata. Based on permutation importance (Figure 2), the factors with contributions exceeding 1.5% are Bio6 (59.8%), Hfp (15.7%), Slope2 (7%), Slope3 (3.4%), Slope8 (2.3%), and elevation (1.7%).

**Figure 3:**
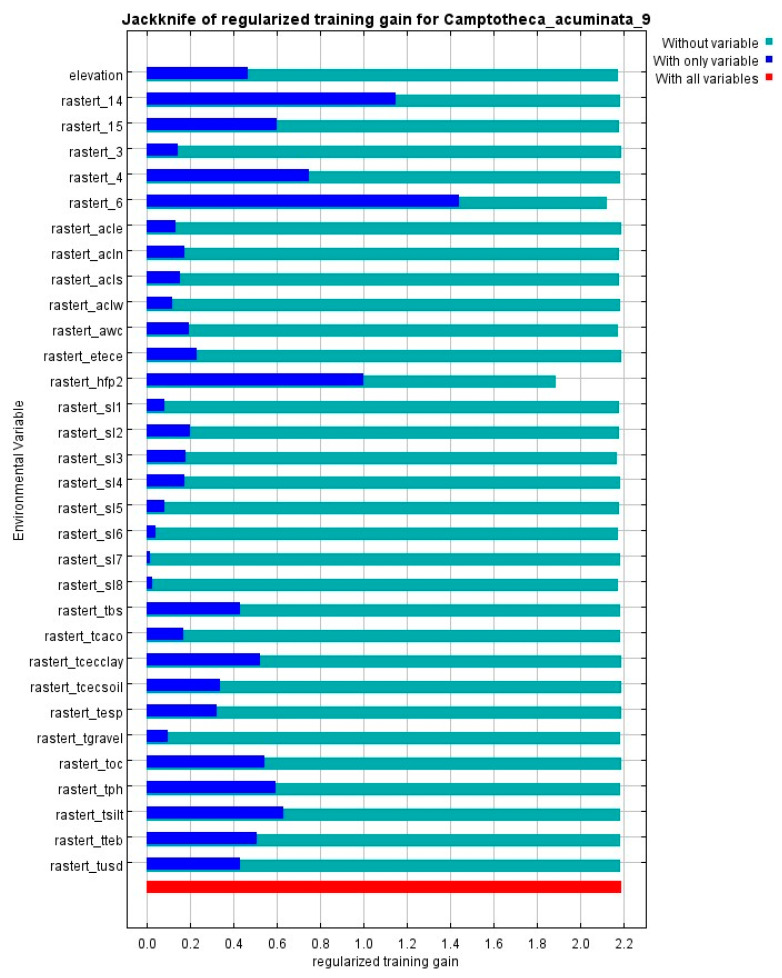
Jackknife method test.

Figure 3 shows the normalized training gain from the jackknife method. Based on the analysis of this figure, it can be observed that under the “only this variable” condition, Slope1, Slope5, Slope6, Slope7, and Slope8 scored less than 0.1, while AspectCLE, AspectCLN, AspectCLS, and AspectCLW scored less than 0.2. These low scores indicate that these variables have a minimal impact on *C. acuminata*’s distribution. Therefore, most topographic factors have little influence on the prediction results. On the other hand, Bio6, Bio14, and Hfp scored over 1.0, indicating that precipitation, temperature, and human activity are the dominant influencing factors.

Based on the combined analysis of contribution rates, permutation importance, and jackknife method tests, the dominant environmental factors selected are Bio14, Hfp, Bio6, Slope2, Slope3, and elevation. The above analysis represents the importance of each factor in the current distribution of *C. acuminata*. The results for the eight future scenarios are generally consistent with those for the present, with contribution rates exceeding 80% still dominated by Bio14, Hfp, Bio6, and Slope3. The permutation importance values, also exceeding 80%, remain dominated by Bio14, Bio6, Hfp, Slope2, Slope3, and elevation. The results from the jackknife method show no significant differences.

### 2.2. MaxEnt Model Accuracy Evaluation for Predicting the Distribution of C. acuminata

The model accuracy was tested using the Area Under the ROC Curve (AUC). After 10 iterations, the maximum test AUC value was selected as the prediction result. In both modern and future climate scenarios, under nine different conditions without human activity influence and nine scenarios with human activity impact, the AUC values of all 18 *C. acuminata* model predictions were greater than 0.9 (Figure S1). This indicates that the model fits well and the prediction results are highly accurate.

### 2.3. Response of Dominant Environmental Factors Based on MaxEnt Model Prediction Results

To further explore the response relationships of dominant environmental factors on the growth of *C. acuminata*, the MaxEnt model was used to derive the response curves for the dominant individual environmental factors: Bio14, Hfp, Bio6, Slope2, Slope3, and elevation. These curves illustrate how the probability of species presence changes with the enhancement of each environmental variable while controlling for other variables by maintaining the average sample value of each.

**Figure 4:**
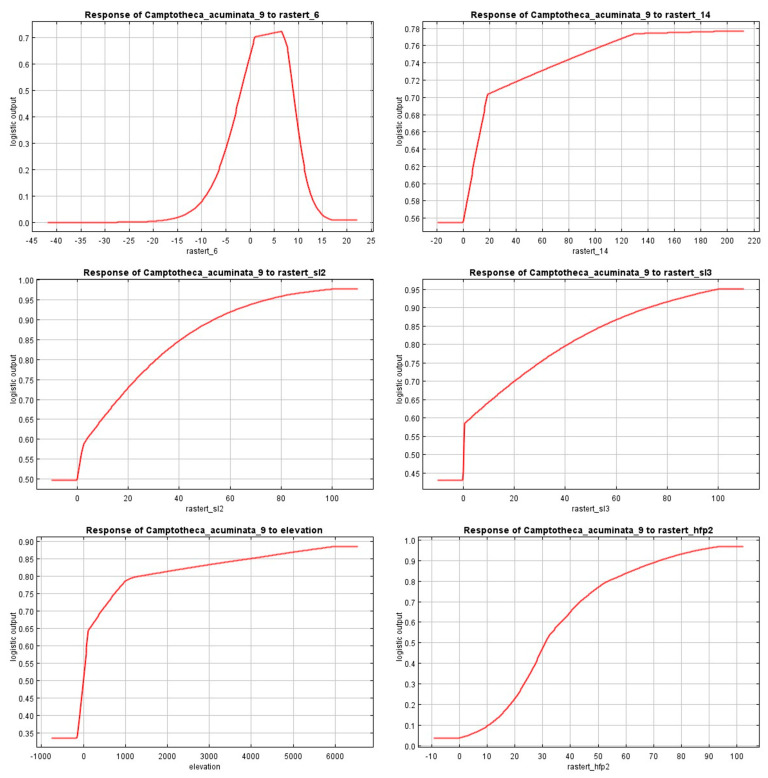
Response of major environmental factors.

**Figure 5:**
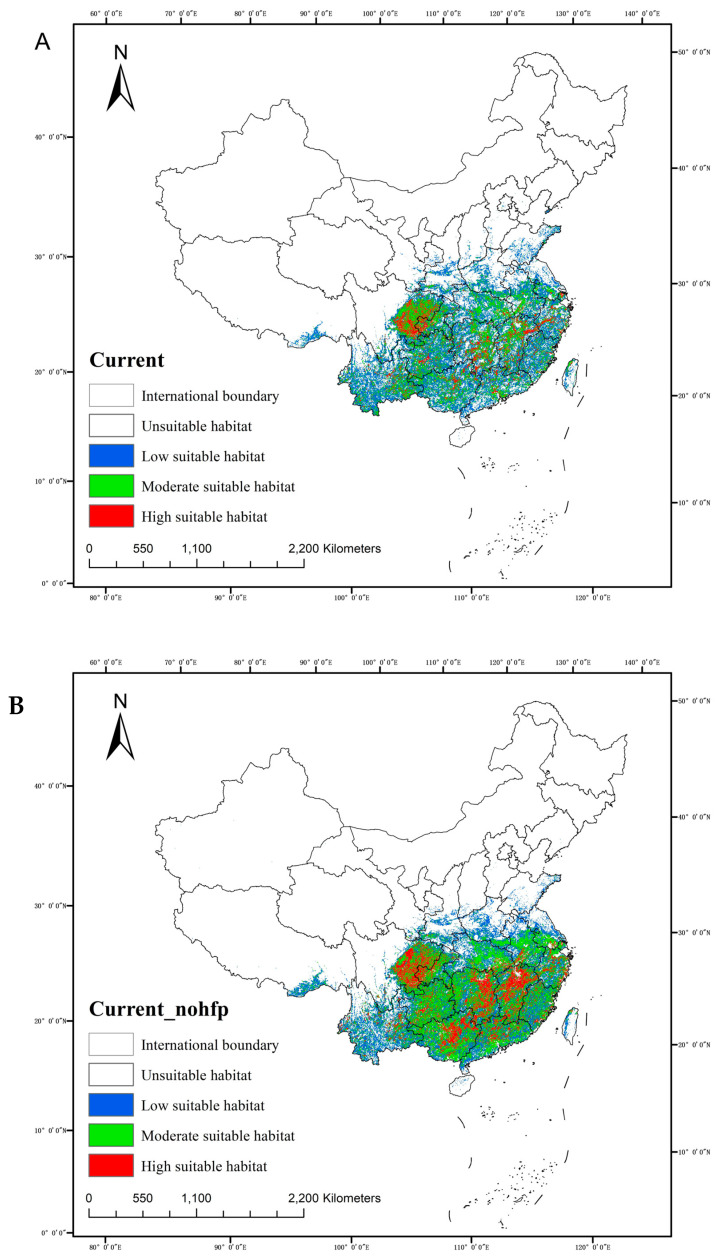
(**A**) The current distribution of suitable areas for *C. acuminata*; (**B**) the current distribution of suitable areas for *C. acuminata* without Global Human Footprint data.

As shown in Figure 4, the probability of *C. acuminata* presence fluctuates significantly with changes in environmental variables. Bio6 is unfavorable for *C. acuminata* growth when it is below −20 °C. As the temperature gradually increases, the probability of presence rises steadily and at an accelerating rate until it reaches around 1 °C, after which the increase slows. The probability peaks at 7 °C and then rapidly declines. Bio14 remains stable when below 0 mm. As the precipitation increases, the probability of presence rises, slowly increasing until it reaches around 18 mm, where it rises more steadily. The probability peaks at 130 mm and then stabilizes. Hfp is unfavorable for growth when it is below 0. As the human activity factor increases, the probability of presence rises steadily and the rate accelerates until around 30. Afterward, the rate of increase slows, peaking at 93 and then stabilizing. Slope 2 remains stable when below 0.5%, but as the slope increases, the probability of presence rises steadily with a gradually decreasing rate. The probability peaks at around a 2% slope and then stabilizes. Slope 3 remains stable below 2%, and as the slope increases, the probability of presence rises sharply. At around 2.1%, the increase slows, and the probability peaks at 5%, after which it stabilizes. Elevation shows a stable minimum below 0 m, then increases sharply, with the probability rising steadily as elevation increases. At approximately 100 m, the rise slows, and at 1000 m, it further slows before reaching a peak at 6000 m. The response curves for *C. acuminata* presence probability with respect to the six dominant environmental factors are divided by a threshold of 0.7. The range where the presence probability exceeds 0.7 represents the suitable environmental conditions for *C. acuminata* growth. According to Figure 5, the suitable range for Bio6 is between 1 °C and 7 °C; Bio14 is between 18 mm and 210 mm; Hfp is between 43 and 103; Slope2 is between 0.74% and 2%; Slope3t is between 2.6% and 5%; and elevation is between 400 m and 6500 m.

### 2.4. Prediction of the Potential Distribution Area of C. acuminata Under Different Climate Scenarios

#### 2.4.1. Current Geographical Distribution

Based on the natural breaks method, the currently suitable habitat areas for *C. acuminata* were divided into four categories: unsuitable habitat areas, low suitable habitat areas, moderate suitable habitat areas, and high suitable habitat areas.

As shown in Figure 5A, the suitable habitat areas for *C. acuminata* are primarily distributed south of the Yangtze River Basin, with the main distribution range spanning 101.625° E to 117.721° E and 24.609° N to 27.522° N. Based on area calculations, the total geographic distribution of *C. acuminata* is 157.96007 × 10^4^ km^2^, accounting for 17.16% of China’s total land area. Among this, the highly suitable habitat areas account for 1.84%, moderate suitable habitat areas account up 6.31%, and low suitable habitat areas account for 9.01%.

The highly suitable habitat areas are widely distributed in the southeastern part of Sichuan and the western part of Chongqing, although not in contiguous patches. They also appear in a linear distribution in the western part of Jiangxi, eastern Zhejiang, and Hunan. Additionally, some highly suitable habitat areas are found in Guizhou and Guangxi. These areas are scattered in Yunnan, Hubei, Guangdong, Anhui, and Fujian.

Moderately suitable habitat areas are concentrated in the eastern part of Sichuan and the western part of Chongqing, with large continuous distributions in eastern Yunnan, Guangxi, Guangdong, Guizhou, eastern Hunan, eastern Hubei, Jiangxi, and Anhui. In contrast, Fujian, Zhejiang, Shaanxi, Henan, Jiangsu, and Taiwan only have scattered distributions.

Low suitable habitat areas are mainly distributed south of the Yangtze River, on the periphery of the Sichuan–Chongqing Basin, and in the middle and lower reaches of the Yangtze River. These areas are widely spread and extend northward to Shaanxi, Henan, and Shandong. It is worth noting that the southeastern parts of Tibet, including Linzhi and Shannan, also have distributions, possibly due to the lower elevation and the subtropical monsoon climate, similar to the climate of most distribution areas.

Due to the inclusion of additional factors such as human activity and topography in this study, which were not considered by previous research [19], a simulation was conducted without the human activity factor to explore its impact on the distribution of *C. acuminata* (Figure 5B). The results show that, without human activity influence, the distribution of suitable habitat areas for *C. acuminata* becomes more concentrated and continuous, rather than fragmented. Both the high and moderate suitable habitat areas significantly increased.

It is evident that the distribution area has not changed significantly, but the areas of highly and moderately suitable habitats have increased substantially. Based on area calculations, in the absence of human activity factors, the total geographical distribution of *C. acuminata* is 184.94791 × 10^4^ km^2^, accounting for 20.09% of China’s total land area. Among this, the highly suitable habitat areas account for 3.46%, moderate suitable habitat areas make up 9.25%, and low suitable habitat areas represent 7.38%. High-suitable habitat areas are primarily distributed in eastern Sichuan, western Chongqing, Guangxi, eastern Hunan, and Jiangxi. Moderately suitable habitat areas are widely distributed in eastern Sichuan, southeastern Hubei, southern Henan, southern Jiangsu, southern Anhui, and areas south of the Yangtze River. Low suitable habitat areas have not expanded much; their area in Shandong has decreased, while there is a slight increase in southern Tibet. Overall, the subtropical and tropical monsoon climate regions south of the Yangtze River in China are more suitable for *C. acuminata* growth, with relatively abundant precipitation and suitable temperature conditions. Without considering the impact of human activity, these two regions are primarily the high and moderately suitable habitats for *C. acuminata*.

With the human activity factor included, the distribution of *C. acuminata* becomes more fragmented, suggesting that deforestation and logging due to human activities have caused significant damage to wild *C. acuminata* resources. Similar impacts of human activity on the distribution of other species have been observed [20]. In terms of total area, the presence of human activity factors has led to a reduction in suitable habitat areas to 26.98784 × 10^4^ km^2^, indicating a negative correlation between human activity and the distribution area of *C. acuminata*.

#### 2.4.2. Potential Geographical Distribution in Future Periods

This study selected four RCP scenarios and two future periods. According to Table 1, the total suitable habitat area for *C. acuminata*, except for RCP8.5 (2050s), shows varying degrees of reduction. Moreover, the locations of suitable habitats exhibit different degrees of spatial migration compared to the present. In the 2050s, the proportion of *C. acuminata*’s total suitable habitat area in China under the RCP2.6, RCP4.5, RCP6.0, and RCP8.5 scenarios will account for 16.81%, 15.95%, 16.33%, and 17.27% of the country’s total land area, respectively. However, by the 2070s, the potential total suitable habitat area will be significantly reduced compared to the 2050s, with the percentages being 16.35%, 15.91%, 15.70%, and 16.86%, respectively.

As shown in Figure S2, in the 2050s, under different greenhouse gas emission scenarios, the area of low suitable habitat for *C. acuminata* decreases in the following order: RCP8.5 > RCP2.6 > RCP6.0 > RCP4.5, with areas of 160.44444 × 10^4^ km^2^, 156.17708 × 10^4^ km^2^, 151.73264 × 10^4^ km^2^, and 148.16146 × 10^4^ km^2^, respectively. The area of moderate suitable habitat decreases in the following order: RCP8.5 > RCP2.6 > RCP6.0 > RCP4.5, with areas of 60.67014 × 10^4^ km^2^, 57.74826 × 10^4^ km^2^, 55.80556 × 10^4^ km^2^, and 53.71007 × 10^4^ km^2^, respectively. The area of high suitable habitat decreases in the following order: RCP2.6 > RCP8.5 > RCP6.0 > RCP4.5, with areas of 17.79514 × 10^4^ km^2^, 17.67361 × 10^4^ km^2^, 16.03819 × 10^4^ km^2^, and 15.12153 × 10^4^ km^2^, respectively. In this period, except for the RCP8.5 scenario, where high suitable habitats increase compared to the present, the areas of suitable habitats in all four scenarios decrease to varying degrees.

In the 2070s, under different greenhouse gas emission scenarios, the area of low suitable habitat for *C. acuminata* decreases in the following order: RCP8.5 > RCP2.6 > RCP4.5 > RCP6.0, with areas of 156.6684 × 10^4^ km^2^, 151.85069 × 10^4^ km^2^, 147.76563 × 10^4^ km^2^, and 145.88368 × 10^4^ km^2^, respectively. The area of moderate suitable habitat decreases in the following order: RCP8.5 > RCP2.6 > RCP4.5 > RCP6.0, with areas of 59.42188 × 10^4^ km^2^, 57.41319 × 10^4^ km^2^, 55.86285 × 10^4^ km^2^, and 55.18403 × 10^4^ km^2^, respectively. The area of high suitable habitat decreases in the following order: RCP4.5 > RCP6.0 > RCP8.5 > RCP2.6, with areas of 19.07465 × 10^4^ km^2^, 18.06076 × 10^4^ km^2^, 17.68576 × 10^4^ km^2^, and 16.34722 × 10^4^ km^2^, respectively.

Under the same greenhouse gas emission scenarios but different periods, the area of low suitable habitat for *C. acuminata* in the 2070s decreases in all four carbon emission scenarios compared to the 2050s. The largest reduction is observed under the RCP6.0 scenario, with a decrease of 7.25 × 10^4^ km^2^, while the smallest reduction is in the RCP8.5 scenario, with a decrease of 2.53993 × 10^4^ km^2^. For the moderately suitable habitat, the area under the RCP4.5 scenario in the 2070s increases compared to the 2050s, while in the RCP2.6, RCP6.0, and RCP8.5 scenarios, the area in the 2070s slightly decreases compared to the 2050s. The situation for a highly suitable habitat follows a similar trend to that of a moderately suitable habitat. It is important to note that under the RCP6.0 scenario, the area shows a large fluctuation. In the 2050s, the area decreased by 6.22744 × 10^4^ km^2^ compared to the present and by 5.84896 × 10^4^ km^2^ in the 2070s. Under the RCP4.5 scenario, the highly suitable habitat area in the 2050s was lower than the current level, with a reduction of 1.8125 × 10^4^ km^2^. However, the area increases by 3.95312 × 10^4^ km^2^ in the 2070s.

The overall trend of suitable habitat areas shows that, except for the RCP8.5 scenario, where the total area of suitable habitats first increases and then decreases, the total area of suitable habitats continues to decrease and reaches its minimum value under the RCP2.6, RCP4.5, and RCP6.0 scenarios. In the RCP8.5 scenario, the area initially increased by 2.48436 × 10^4^ km^2^ in the 2050s but then decreases by 3.77604 × 10^4^ km^2^ in the 2070s. Under the RCP2.6 scenario, the total area of suitable habitats for *C. acuminata* in the 2050s decreases by 1.783 × 10^4^ km^2^ compared to the present, and in the 2070s, it further decreases by 4.32639 × 10^4^ km^2^ compared to the 2050s. In the RCP4.5 scenario, the total area of suitable habitats in the 2050s is reduced by 9.79862 × 10^4^ km^2^ compared to the present, and in the 2070s, it further decreases by 0.39583 × 10^4^ km^2^ compared to the 2050s. In the RCP6.0 scenario, the area decreases by 6.22743 × 10^4^ km^2^ in the 2050s and continued to decrease by 5.84896 × 10^4^ km^2^ in the 2070s.

As shown in Figure S3, in the absence of human activity interference, the total suitable habitat area for *C. acuminata* increases under all scenarios in the 2050s. However, in the 2070s, except for the RCP8.5 scenario, where the suitable habitat area increases compared to the 2050s, the suitable habitat areas under the RCP2.6, RCP4.5, and RCP6.0 scenarios all decrease compared to the 2050s.

This suggests that in the 2050s, the increase in temperature and humidity should have caused an expansion in the total suitable habitat area for *C. acuminata*. However, due to the interference of future human activities and other factors, the total suitable habitat area actually decreases in the 2050s. Compared to the 2050s, the suitable habitat area in the 2070s decreases across all scenarios. This indicates that, with climate change, the distribution range of suitable habitats for *C. acuminata* is expected to shrink as greenhouse gas emissions increase over time.

#### 2.4.3. Dynamic Changes in Suitable Habitats of *C. acuminata* Under Different Future Climate Scenarios

As shown in Figure S4, under the RCP2.6 scenario, the total suitable habitat area for *C. acuminata* in the 2050s is 156.17708 × 10^4^ km^2^, which is a reduction of 1.13% compared to the present. The primary reductions are observed in moderate and low suitable habitat areas, mainly in Sichuan, Anhui, Jiangsu, Zhejiang, and Fujian. However, the highly suitable habitat areas show slight expansion in Guangzhou, Taiwan, Hunan, and Jiangxi.

In the 2070s, compared to the 2050s, the total suitable habitat area decreases by 2.77%, with a total area of 151.85069 × 10^4^ km^2^, and all three habitat types see reductions. The areas with the most significant changes are in southern Tibet and central–southern Yunnan. Nearly two-thirds of the low-suitable habitat area in Tibet disappears, and almost half of the low-suitable habitat in Yunnan vanishes. Additionally, part of the highly suitable habitat in the southeastern part of Sichuan shifts to a moderately suitable habitat. However, the moderate and low suitable habitat areas in Hunan and Jiangxi, which were reduced in the 2050s, show some recovery. As more proactive measures are taken to reduce greenhouse gases, significant reductions in greenhouse gas emissions will provide more favorable conditions for *C. acuminata* growth. This suggests that human activity does not only have a negative impact, such as deforestation and logging but can also have positive effects when greenhouse gas emissions are actively controlled.

Under the RCP4.5 scenario, the total suitable habitat area for *C. acuminata* in the 2050s decreases by 3.87%, reaching 148.16146 × 10^4^ km^2^. All three habitat types show reductions, with the moderate suitable habitat experiencing the largest decrease, followed by the low suitable habitat. The primary areas of reduction are the highly suitable habitat in the southeastern part of Sichuan, and the low suitable habitat in Henan, Shandong, southern Tibet, and northern Hubei. The reductions in all three habitat types are larger than those in the RCP2.6 scenario.

By the 2070s, the expansion of suitable habitats for *C. acuminata* significantly decreases, while the areas of reduction become more widespread. Both the low and moderate suitable habitat areas are further reduced compared to the 2050s, while the high suitable habitat area increases. Areas previously contracted in the 2050s, such as those in Shandong, expanded back almost completely. Additionally, the highly suitable habitat areas that were originally reduced in the 2050s are now expanding again, and in some regions, the expansion is even more concentrated than in the present, particularly in areas where eastern Sichuan meets western Chongqing and in southern Hunan. Other provinces where expansion occurs include Shandong, Henan, and Shanxi. The reduction areas are found in the central and southern parts of Yunnan, similar to the reductions observed in the RCP2.6 scenario for the same year.

Under the RCP6.0 scenario, the total suitable habitat area for *C. acuminata* in the 2050s is 151.73264 × 10^4^ km^2^, which is a decrease of 3.94% compared to the present. All three habitat types show a reduction, with the low and moderate suitable habitat areas experiencing larger decreases, while the high suitable habitat area shows a smaller reduction. The expansion is minimal, mainly occurring in Hainan, where the number of suitable habitat areas has increased. The low suitable habitat area has primarily shrunk in northwestern Hubei, Hunan, Jiangxi, Anhui, and Zhejiang. The moderately suitable habitat decreases across southern regions of the Yangtze River Basin, with a larger range of reduction, though the unit area decrease is smaller. Additionally, a small patch of highly suitable habitat appears in northern Taiwan.

By the 2070s, the total suitable habitat area decreases further, with a total area of 145.88368 × 10^4^ km^2^, which is a reduction of 3.84% compared to the 2050s. Both the low and moderate suitable habitat areas continue to shrink, while the high suitable habitat area shows a slight increase. The low suitable habitat area experiences the most significant reduction, mainly in southern Tibet, central–southern Yunnan, Guangdong, Jiangxi, Fujian, Zhejiang, and Hainan. The area in Hainan that had expanded in the 2050s nearly disappeared by the 2070s. The number of suitable habitat areas slightly increases in the Hunan and Jiangxi provinces.

Under the RCP8.5 scenario, the total suitable habitat area for *C. acuminata* in the 2050s is 160.44444 × 10^4^ km^2^, which is an increase of 1.57% compared to the present, making it the scenario with the largest increase in suitable habitat area. Except for the reduction in low suitable habitat, all other areas expanded. The primary expansions occurred in moderately suitable habitats, including regions in Guangxi, Guangdong, central Shaanxi, and southern Henan. High suitable habitats slightly expanded in eastern Sichuan, central Guangdong, and northern Taiwan, while other areas experienced scattered expansions. The reduction in low suitable habitat was not significant, with a slight increase observed in Hainan.

By the 2070s, the total suitable habitat area for *C. acuminata* decreases to 156.6684 × 10^4^ km^2^, which is a reduction of 2.35% compared to the 2050s. Except for the highly suitable habitat, which showed no area change, both moderate and low suitable habitats decrease to some extent. The main reduction occurred in the low suitable habitat, with the area that had expanded in Hainan in the 2050s shrinking back to the current suitable habitat area. The highly suitable habitat in eastern Sichuan became more concentrated in the 2070s compared to the 2050s, but reductions in highly suitable habitats were observed in other areas, such as northern Taiwan. The reduction in moderate suitable habitat was mainly in eastern Hubei and Taiwan.

#### 2.4.4. Migration Map of the Geometric Centre of Suitable Habitats in Different Periods

**Figure 6:**
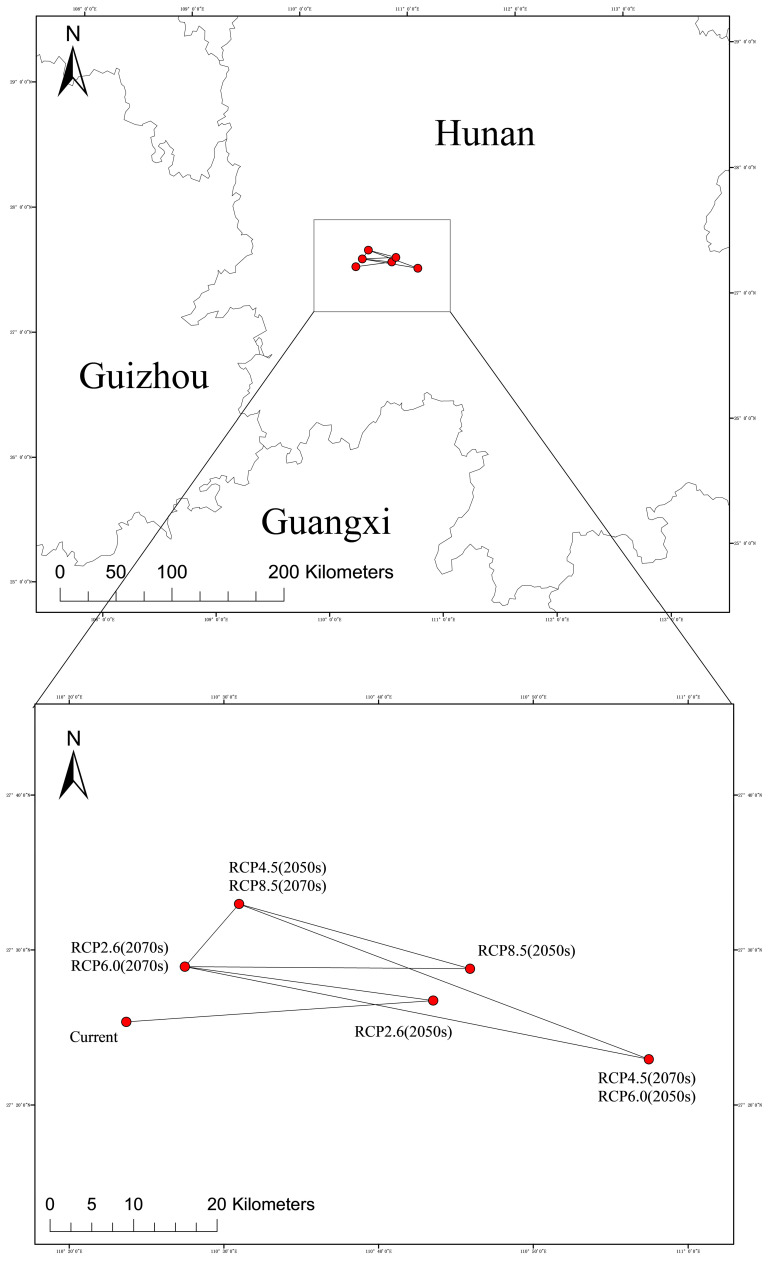
Centroid migration in suitable areas at different stages.

As shown in Figure 6, the potential distribution center of *C. acuminata* is located in Zhongfang County, Huaihua City, Hunan Province (27.42° N, 110.39° E). Under the RCP2.6-2050s climate scenario, the distribution center is located in Longhui County, Shaoyang City, Hunan Province (27.44° N, 110.73° E). In the RCP2.6-2070s climate scenario, the distribution center shifts to Xupu County, Huaihua City, Hunan Province (27.48° N, 110.46° E). For the RCP4.5-2050s climate scenario, the distribution is in Xupu County, Huaihua City, Hunan Province (27.55° N, 110.51° E). In the RCP4.5-2070s scenario, the distribution center moves to Longhui County, Shaoyang City, Hunan Province (27.38° N, 110.96° E). Under the RCP6.0-2050s scenario, the distribution center remains in Longhui County, Shaoyang City (27.38° N, 110.96° E), while in the RCP6.0-2070s scenario, it shifts to Xupu County, Huaihua City (27.48° N, 110.46° E). In the RCP8.5-2050s and RCP8.5-2070s scenarios, the distribution centers are located in Longhui County, Shaoyang City, Hunan Province (27.48° N, 110.77° E) and Xupu County, Huaihua City, Hunan Province (27.55° N, 110.52° E), respectively.

In the 2050s, under the RCP2.6 scenario, the centroid of *C. acuminata* shifts 33.6295 km northeast compared to the present. Under the RCP4.5 scenario, the centroid shifts 18.6837 km northeast. In the RCP8.5 scenario, the centroid shifts 38.0858 km northeast. In contrast, under the RCP6.0 scenario, the centroid moves southeast, with the largest shift being 56.4463 km, which is different from the other scenarios.

In the 2070s, under the RCP2.6 scenario, the centroid shifts 27.0088 km northwest compared to the 2050s. Under the RCP6.0 scenario, the centroid moves 50.5842 km northwest. In the RCP8.5 scenario, the centroid shifts 25.8540 km northwest compared to the 2050s. However, under the RCP4.5 scenario, the centroid shifts southeast by 48.7029 km compared to the 2050s, showing a different direction than the other scenarios.

Except for the RCP6.0 scenario, where the centroid first moves southeast and then northwest, in all other scenarios, the centroid first moves northeast and then northwest.

## 3. Discussion

### 3.1. Errors in Distribution Point Data and Environmental Variable Selection

**Figure 7:**
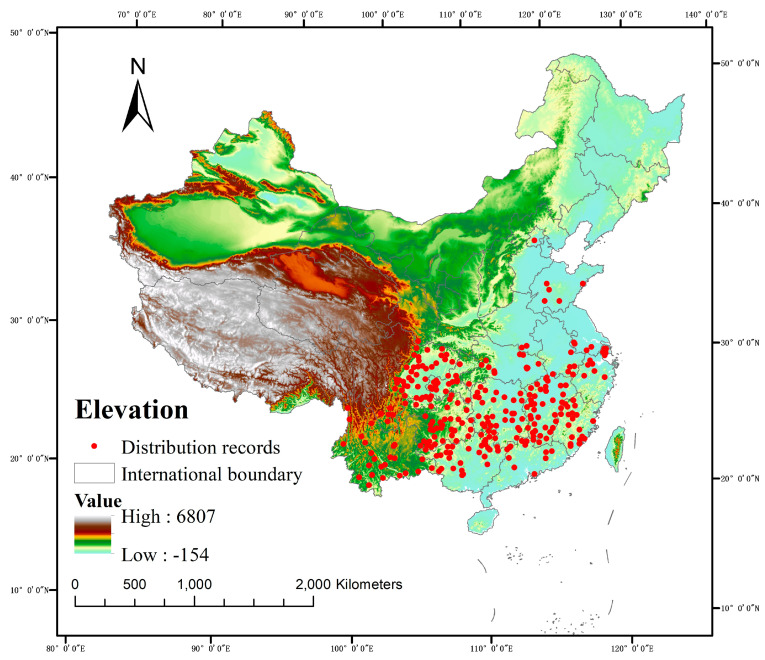
Current distribution of *C. acuminata*.

This study collected 1008 distribution point data records for *C. acuminata* (Figure 7), sourced from the National Specimen Information Infrastructure, the China Virtual Herbarium, and the Global Biodiversity Information Facility. Overall, although the data collection covered a wide range, some errors inevitably occurred while gathering and organizing the distribution point data. Some errors might have been caused by factors such as the passage of time or illegible handwriting, which resulted in inaccurate recording of distribution points. Environmental variables significantly influence the potential distribution of species, with factors such as topography and human activity independently affecting species distribution. Studies by Vaughan and Liu et al. indicate that human activities, such as population density and hydropower projects, can potentially influence species distribution [21,22]. Research by Luo et al. further shows that the potential suitable habitats of species in the *Impatiens* genus are significantly affected by human activities [17]. Therefore, this study aimed to collect as many distribution points as possible to minimize errors caused by these factors. Environmental variables significantly influence the potential distribution of species. In this study, 50 environmental factors were selected as predictors for the environmental variables of *C. acuminata*. Although the selection of climate variables was comprehensive and the resolution was high, making the prediction results relatively reliable, other factors such as competition for living space among invasive plants, natural disasters, pests, diseases, and damage from wild animals can also affect species distribution to some extent. Future studies should consider incorporating additional influencing factors to explore the potential distribution areas of species further. These results should be compared with previous predictions to identify the optimal combination of environmental variables, thus enhancing the accuracy of the model’s predictions [23].

The predictive ability of the MaxEnt model has been validated in several studies, which show that under the same conditions, the model demonstrates higher prediction accuracy, surpassing other models [24,25,26,27,28,29,30,31]. In this study, the suitable habitat areas for *C. acuminata* were predicted using the MaxEnt model, with AUC values exceeding 0.9, indicating that the prediction results have high accuracy. Improvements can be made in future research from multiple perspectives to enhance the model’s predictive accuracy and achieve better results. Adjusting parameters to suit the predictions for different species can smooth the model’s complexity, improve prediction outcomes, and more accurately reflect the species’ response to environmental changes [32]. By combining multiple models for simulating and predicting the potential distribution areas of species, and conducting a comparative analysis of the prediction results, the strengths of each model can be leveraged. This approach integrates the advantages of different models, ultimately selecting the most optimal prediction outcome and further enhancing the accuracy of individual model predictions.

### 3.2. Changes in the Suitable Distribution Area of C. acuminata in Different Periods

Based on the MaxEnt model, this study predicted the potential suitable habitat distribution of *C. acuminata* in China. The results show that the current potential distribution of *C. acuminata* is primarily concentrated in the Sichuan Basin and surrounding areas, the Yunnan Plateau, the middle and lower reaches of the Yangtze River, and the South China region, all located south of the Qinling–Huaihe line. The predicted results are consistent with the actual distribution of *C. acuminata* in China and previous research findings [33]. Additionally, the AUC value of the ROC curve is 0.962, indicating that the prediction results are reliable. By simulating and predicting the distribution range of *C. acuminata* in different periods, this study also infers its future change trend.

With the increasing greenhouse gas emissions and the gradual intensification of the greenhouse effect in the future, the distribution of suitable habitats for *C. acuminata* is expected to undergo corresponding changes in both area and spatial patterns. Feng et al.’s study indicates that under the RCP4.5 and RCP8.5 climate change scenarios in the 2020s, 2050s, and 2080s, the suitable habitat for *C. acuminata* will moderately increase in future climates. Currently, the high-suitability habitats of *C. acuminata* are located at the junction of Guizhou and Guangxi, Hunan and Guangdong, as well as Fujian, Jiangxi, and Zhejiang [28]. In the absence of human activity factors, under different greenhouse gas emission scenarios but the same time period, the total suitable habitat area in the 2050s first increases significantly, then the increase slows down as greenhouse gas concentrations rise. The largest increase in the area occurs under the RCP6.0 scenario. By the 2070s, the total suitable habitat area decreases initially and then expands, with the largest expansion occurring under the RCP8.5 scenario. The expansion area increases from RCP2.6 to RCP6.0 in the 2050s, showing a positive correlation with increasing greenhouse gas concentrations. As greenhouse gas concentrations rise, the reduction area decreases in the 2070s from RCP2.6 to RCP6.0. When considering the same greenhouse gas emission scenario but different time periods, the total suitable habitat area changes over time, increasing and decreasing. The RCP8.5 scenario is an exception, showing a continuous increase in area. The expansion area decreases over time in all scenarios except for RCP8.5, where it increases. Similarly, the reduction area increases over time in all scenarios except for RCP8.5, where it decreases.

Under no human activity factors, RCP6.5 and RCP8.0 are more suitable for *C. acuminata*’s future growth. However, under the influence of human activity factors, RCP2.6 and RCP8.0 are more suitable for future growth. Under different future RCP scenarios, the movement of the centroid varies slightly. From the present to the 2050s, the centroid shifts eastward in all four scenarios, with the RCP4.5 scenario specifically shifting towards the northeast. From the 2050s to the 2070s, except for the RCP4.5 scenario, where the centroid continues to shift eastward, the centroid in the other scenarios, particularly RCP6.0, moves westward. In most scenarios, as greenhouse gas concentrations increase and time progresses, the centroid of *C. acuminata* migrates toward higher latitudes. The study by Varol and Tekin et al. found that, in the future, the suitable habitats of many species in low-latitude regions will decrease, while suitable habitats in high-latitude regions will increase. Overall, the suitable habitats will shift toward higher latitudes [34,35,36].

### 3.3. Ecological Characteristics Affecting the Distribution Area of C. acuminata

The results of this study indicate that, among the 32 selected environmental factors, temperature, precipitation, human activity, and slope all have a certain degree of influence on the geographical distribution of *C. acuminata*. According to the three testing methods, precipitation is the most important factor, followed by temperature and then human activity. The main environmental factors influencing the growth of *C. acuminata* are Bio14, Hfp, Bio6, Slope2, Slope3, and elevation, with their suitable ranges being 18–210 mm, 43–103, 1–7 °C, 16–100%, 20–100%, and 400–6500 m. It can be concluded that the environment suitable for *C. acuminata* growth is warm and humid, with abundant precipitation, higher altitudes, and low human activity, which is consistent with Zhang’s [1] research on the ecological habits of *C. acuminata*. As mentioned earlier, human activity is negatively correlated with the distribution area of *C. acuminata*, meaning that the greater the human activity intensity, the less suitable the environment for *C. acuminata* growth. The unprecedented pressure caused by human activities is a major external factor driving the endangerment of *C. acuminata*. It is well known that human activities are mainly concentrated in low-altitude areas. Since *C. acuminata* typically grows in low-altitude, warm, and humid regions, activities such as deforestation, land reclamation, and logging not only directly destroy *C. acuminata* populations but also lead to consequences such as soil desiccation, which indirectly damages the species’ habitat and further reduces the viability of the populations. Additionally, *C. acuminata* is an important medicinal resource plant in the local area, and activities such as seed collection, leaf picking, and bark stripping every year also impact the growth and reproduction of the population, severely threatening the survival of *C. acuminata* populations [2].

Additionally, a large area of moderately suitable habitat in the Sichuan Basin is expected to transition into a highly suitable habitat under the scenario of increasing greenhouse gas concentrations in the future. The reason for this shift, with highly suitable habitats surrounding low suitable habitats, is likely due to the intensified greenhouse effect. In the Sichuan Basin, climate change is expected to primarily manifest as rising temperatures, increased rainfall (mainly concentrated between June and September), and more extreme rainfall events. Moreover, the basin’s soil, rich in nitrogen and composed of loamy clay, along with abundant surface and groundwater, creates a more favorable environment for C. acuminata growth. This combination of factors makes the Sichuan Basin increasingly suitable for the species’ growth in the future [37,38]. Other factors, such as soil and aspect, have a relatively small impact on the distribution of *C. acuminata*. Among the aspect factors, the one with a slightly larger contribution is AspectCIS, with a range of 700° to 3400°, indicating that *C. acuminata* is more suitable for growing in areas with larger slopes. Among soil factors, the one with a slightly higher contribution is T_PH_H2O, which refers to the soil reaction of topsoil. This factor mainly describes the soil pH level. According to the response curve, *C. acuminata* is suitable for growth in soils with a pH range of 3.6 to 6.9.

Focused protection measures should be implemented to ensure the conservation of *C. acuminata*. This includes forest protection policies, a strict ban on land reclamation and grazing, establishing key protected areas, and setting up observation stations to monitor and assess the growth status of wild *C. acuminata* populations, allowing for timely and appropriate conservation actions. Additionally, efforts should be made to strengthen research on artificial cultivation and introduction techniques for *C. acuminata*, expanding the cultivation scale to meet the market demand for camptothecin. Protected areas should be established for effective conservation and management based on the future distribution of suitable habitats.

## 4. Materials and Methods

### 4.1. Collection and Processing of Species Distribution Data

The geographical information data of *C. acuminata* were sourced from the National Specimen Information Infrastructure (http://www.nsii.org.cn/), the China Virtual Herbarium (https://www.cvh.ac.cn/index.php), and the Global Biodiversity Information Facility (https://www.gbif.org/) [39]. Specimen data from the 19th century to the present were selected, with duplicates removed, and unclear records and cultivated records filtered out. Since the spatial resolution used in this study is 2.5 arc-minutes (approximately 4.5 km), a buffer radius of 3 km was set. When two distribution points fall within the same buffer zone, only one point is retained. The data were processed in EXCEL to create a CSV file containing only three columns, consisting of species name, longitude, and latitude, facilitating the subsequent construction of the MaxEnt model.

### 4.2. Environmental Factors Data

Climate factors: The 19 contemporary and future bioclimatic variables were sourced from the WorldClim database (WorldClim-Global Climate Data: http://www.worldclim.org/) [40]. Future climate variables are derived from the projections of global climate change presented in the IPCC’s Fifth Assessment Report. The atmospheric circulation model used is the BCC_CSM model developed by the National Climate Center of China. The RCP2.6, RCP4.5, RCP6.0, and RCP8.5 scenarios represent four different levels of carbon emission scenarios for future climate predictions, with the numbers indicating the radiative forcing levels ranging from 2.6 Wm⁻^2^ to 8.5 Wm⁻^2^ by 2100 [41]. A total of eight climate scenario combinations are considered: RCP2.6-2050, RCP2.6-2070, RCP4.5-2050, RCP4.5-2070, RCP6.0-2050, RCP6.0-2070, RCP8.5-2050, and RCP8.5-2070.

Soil factors: The 16 soil variables were obtained from the Harmonized World Soil Database (http://www.fao.org/soils-portal/soil-survey/soil-maps-and-databases/harmonized-world-soil-database-V12-en; 20 March 2023).

Landform factors: Elevation data were obtained from the SRTM elevation data, available at the WorldClim Global Climate Data website (http://www.worldclim.org/). Global slope and aspect data are available from the Harmonized World Soil Database (http://www.fao.org/soils-portal/soil-survey/soil-maps-and-databases/harmonized-world-soil-database-V12-en; 20 March 2023).

Human activity factors: The human activity intensity data were sourced from the Global Human Footprint v2 (1995–2004) dataset provided by the International Earth Science Information Center. All environmental factors details are shown in Table 2.

In this study, the MaxEnt 3.4.1 model software and ArcGIS 10.2 were used for data analysis. First, all data were clipped according to the 1:1,000,000 administrative vector map of China. Using ArcGIS, 50 environmental factor datasets were converted to the ASC format. The environmental factors were further processed by extracting them using the “Toolbox/Spatial Analyst Tools/Extraction/Extract by Mask” tool and resampling them using the “Toolbox/Data Management Tools/Raster/Raster Processing/Resample” tool in ArcGIS 10.2. The final environmental variable raster images had consistent extents and pixel sizes [42], with a uniform resolution of 2.5 min and a geographic coordinate system of GCS_WGS_1984. The map of China was downloaded from the Standard Map Service Website of the National Geomatics Center of China (http://bzdt.ch.mnr.gov.cn/index.html). The map, with the approval number GS (2019) 1822 and a scale of 1:1,000,000, was converted into SHP format for subsequent analysis and mapping.

### 4.3. Model Pre-Run and Evaluation Metrics

To prevent overfitting among environmental variables and ensure the model’s accuracy, preprocessed CSV format distribution data of *C. acuminata* in China and ASC format data of 50 environmental factors were used in MaxEnt for preliminary simulation. A total of 25% of the distribution points were set as the test set, while 75% were used as the training set. The model was run 10 times with repeated simulations. MaxEnt automatically generates a Receiver Operating Characteristic (ROC) curve, with specificity on the x-axis and sensitivity on the y-axis, and calculates the Area Under the ROC Curve (AUC) value. Model validation is typically performed using the ROC curve method, where the AUC value, representing the Area Under the ROC Curve, serves as one of the model evaluation metrics. Since the AUC value is independent of the threshold, it offers high reliability, ranging from [0, 1] [19]. Theoretically, a model with an AUC value between 0.5 and 0.6 has no predictive ability; a model with an AUC value between 0.6 and 0.7 has poor predictive performance; a model with an AUC value between 0.7 and 0.8 has moderate predictive capability; a model with an AUC value between 0.8 and 0.9 has good predictive performance; and when the AUC value exceeds 0.9, the model exhibits extremely high accuracy [20].

### 4.4. Environmental Factor Correlation Analysis

Since environmental variables may be correlated, applying all of them directly to the model could lead to overfitting. To address this, the Multivariate tool in ArcGIS 10.2 was used to conduct multiple linear analyses on the 50 environmental variables. Pearson correlation coefficients (r) were calculated to assess the degree of correlation between climatic and soil variables, and the results were visualized in a heatmap for climate factor correlation analysis. If the correlation between two environmental variables was ≥0.8, the dominant variable with a higher contribution in the pre-simulation was retained [43].

The jackknife method was employed to assess the impact of different variables on the habitat of *C. acuminata* and to select the environmental factors used for distribution prediction. The description of regularized training gain effectively reflects whether the maximum entropy distribution or a uniform distribution better fits the data. In Figure 4, the dark blue bars represent the gain when each variable is used individually, indicating how much each factor contributes to species distribution when considered alone. This also identifies key environmental factors influencing the suitable habitat of *C. acuminata*, allowing for an evaluation of each variable’s impact on species distribution [42,44].

### 4.5. Model Computation

The selected environmental factors and *C. acuminata* distribution data were imported into MaxEnt 3.4.1. In the Basic settings, 25% of the distribution data were set as the test set for accuracy validation, while 75% was used as the training set to drive the model. The Random seed option was checked, the repetition type was set to Subsample, and the model was run 10 times. The output distribution values were saved in logistic format. The Create response curve option was selected to generate curves illustrating how climatic factors influence the predicted occurrence probability. Additionally, the Do jackknife option was enabled to output the contribution rate of each climatic factor. All other parameters were kept at their default settings.

### 4.6. Habitat Suitability Classification

The prediction results generated by MaxEnt were imported into ArcMap, where the simulated ASC files were converted into raster format. The Reclassify tool in ArcGIS (Spatial Analyst Tools—Reclass—Reclassify) was used to classify the habitat suitability into four categories: unsuitable areas (*p* < 0.07), low-suitability areas (0.07 < *p* < 0.2), moderate-suitability areas (0.2 < *p* < 0.5), and high-suitability areas (*p* > 0.5). Different colors were applied to represent these suitability levels, and the area of each suitable habitat category was calculated accordingly. ArcGIS was further used for analyzing and visualizing the prediction results, generating potential distribution maps of *C. acuminata* under current conditions, as well as for 2050 and 2070 under four greenhouse gas concentration scenarios: RCP2.6, RCP4.5, RCP6.0, and RCP8.5.

### 4.7. Dynamic Changes in the Distribution Area of C. acuminata Under Different Future Climate Scenarios

Under future climate change scenarios, the suitable distribution areas of plants will shift, and the area of highly suitable habitats will vary across different climate scenarios. To analyze these changes, the SDM Toolbox in ArcGIS 10.2, which is scripted in Python 3.12 [42], was used to quantify the future changes in the potential distribution area and the shift of the suitable habitat center for *C. acuminata*. The statistical results categorize habitat changes into three types: expansion, stability, and contraction. This study primarily utilizes the SDM Toolbox to assess the variations in the distribution of *C. acuminata* under different climate scenarios compared to the current suitable habitat.

### 4.8. Centroid Migration of Suitable Habitats in Different Periods

By utilizing ArcGIS, the suitable habitat of *C. acuminata* can be treated as an indivisible whole and compressed into a vector centroid, providing a more intuitive and simplified representation. This approach allows for the analysis of habitat evolution trends and the derivation of centroid locations for the current and future suitable areas under four climate scenarios. The position and shifting trend of this geometric center reflect the overall spatial distribution and migration trajectory of *C. acuminata*’s suitable habitat.

## 5. Conclusions

This study innovatively integrated 50 environmental factors, including climate, soil, topography, and human activity, to predict the potential distribution of suitable habitats for *C. acuminata* in China under current and future climate scenarios using the MaxEnt model. The results indicate that the potential suitable habitat area for *C. acuminata* generally shows an expanding trend, although this expansion is limited under the highest greenhouse gas emission scenario (RCP8.5). The suitable habitat area is significantly influenced by human activities, exhibiting a pattern of “initial expansion followed by contraction”, except under the RCP8.5 scenario, where the area shows a trend of “initial expansion and subsequent shrinkage”. Centroid migration analysis reveals that, driven by global warming, the potential suitable habitats for *C. acuminata* are gradually shifting northward. This study not only analyzes the species’ environmental adaptability but also highlights the critical role of human activity in shaping its future distribution patterns, providing scientific support for the conservation, resource management, and planting strategies of C. acuminata under changing environmental conditions. Future research could further integrate long-term field monitoring data to validate the model predictions’ reliability and optimize conservation and management measures.

## Figures and Tables

**Table 1:** Areas of potential distribution under different climate scenarios of *C. acuminata*.

	Period	Climate Scenarios	Low-Level Suitable Area /×10^4^ km^2^ (%)	Moderately Suitable Area /×10^4^ km^2^ (%)	Highly Suitable Area /×10^4^ km^2^ (%)	Total Suitable Area /×10^4^ km^2^
With-Hfp	Current	/	82.91146 (54.72%)	58.11458 (38.36%)	16.93403 (11.18%)	151.51563
	2050s	RCP2.6	80.63368 (51.05%)	57.74826 (36.56%)	17.79514 (11.27%)	157.96007
		RCP4.5	78.09028 (50.00%)	57.41319 (36.76%)	16.34722 (10.47%)	156.17708
		RCP6.0	79.32986 (52.24%)	53.71007 (35.37%)	15.12153 (9.96%)	151.85069
		RCP8.5	72.82813 (49.15%)	55.86285 (37.70%)	19.07465 (12.87%)	148.16146
	2070s	RCP2.6	79.88889 (54.06%)	55.80556 (37.77%)	16.03819 (10.85%)	147.76563
		RCP4.5	72.63889 (47.87%)	55.18403 (36.37%)	18.06076 (11.90%)	151.73264
		RCP6.0	82.10069 (56.28%)	60.67014 (41.59%)	17.67361 (12.11%)	145.88368
		RCP8.5	79.56076 (49.59%)	59.42188 (37.04%)	17.68576 (11.02%)	160.44444
No-Hfp	Current	/	67.90451 (43.34%)	85.16666 (54.36%)	31.87674 (20.35%)	156.66840
	2050s	RCP2.6	72.60069 (39.25%)	82.33507 (44.52%)	33.04514 (17.87%)	184.94791
		RCP4.5	72.20660 (38.41%)	82.22049 (43.74%)	31.87500 (16.96%)	187.98090
		RCP6.0	68.02084 (36.51%)	86.92188 (46.66%)	36.66493 (19.68%)	186.30209
		RCP8.5	72.09722 (37.63%)	81.07639 (42.31%)	35.51910 (18.54%)	191.60765
	2070s	RCP2.6	70.75347 (37.50%)	93.54340 (49.57%)	30.99306 (16.43%)	188.69271
		RCP4.5	69.59375 (35.64%)	84.28125 (43.16%)	30.61285 (15.68%)	195.28993
		RCP6.0	75.45313 (40.90%)	83.92361 (45.49%)	29.36806 (15.92%)	184.48785
		RCP8.5	80.06771 (42.42%)	85.97049 (45.55%)	32.50000 (17.22%)	188.74480

**Table 2:** Ecological and environmental factors used in simulation.

Data Type	Ecological Factor	Units
Climate factor	Mean annual temperature (bio1)	°C
	Mean monthly temperature range (bio2)	°C
	Isothermality (bio3)	/
	Temperature seasonality (bio4)	/
	Max temperature of the warmest month (bio5)	°C
	Min temperature of the coldest month (bio6)	°C
	Temperature annual range (bio7)	°C
	Mean temperature of the wettest quarter (bio8)	°C
	Mean temperature of the driest quarter (bio9)	°C
	Mean temperature of warmest quarter (bio10)	°C
	Mean temperature of coldest quarter (bio11)	°C
	Annual precipitation (bio12)	mm
	Precipitation of wettest month (bio13)	mm
	Precipitation of driest month (bio14)	mm
	Precipitation seasonality (bio15)	mm
	Precipitation of wettest quarter (bio16)	mm
	Precipitation of driest quarter (bio17)	mm
	Precipitation of warmest quarter (bio18)	mm
	Precipitation of coldest quarter (bio19)	mm
Soil factor	Volume percentage of gravel in the topsoil (T_gravel)	%vol.
	Percentage of sand in the topsoil (T_SAND)	% wt.
	Percentage of silt in the topsoil (T_SILT)	% wt.
	Percentage of clay in the topsoil (T_CLAY)	% wt.
	Texture class name and code (T_USDA_TEX_ CLASS)	name
	Cation exchange capacity (T_REF_BULK_DENSITY)	kg/dm^3^
	Percentage of organic carbon in the topsoil (T_OC)	% weight
	Soil reaction of topsoil (T_PH_H2O)	log(H+)
	Cation exchange capacity of the clay fraction in the topsoil (T_CEC_CLAY)	cmol/kg
	Cation exchange capacity in the topsoil (T_CEC_SOIL)	cmol/kg
	Base saturation in the topsoil (T_BS)	%
	Calcium carbonate (lime) content in the topsoil (T_CACO3)	%
	Exchangeable sodium percentage in the topsoil (T_ESP)	%
	Electrical conductivity of topsoil (T_ECE)	dS/m
	Total exchangeable bases in the topsoil (T_TEB)	cmol/kg
	Available water storage capacity in mm/m of the soil unit (AWC)	%
Landform factor	Elevation	m
	Slope	%
	Aspect	°
Human activity factors	Global Human Footprint	/

## Data Availability

No new data were created or analyzed in this study.

## Supplementary Materials

The following supporting information can be downloaded at https://www.mdpi.com/article/10.3390/plants14101466/s1: Figure S1: ROC curve of the MaxEnt model; Figure S2: Distribution of *C. acuminata* in the 2050s and 2070s under different greenhouse gas concentration scenarios; Figure S3: Distribution of *C. acuminata* in the 2050s and 2070s under different greenhouse gas concentration scenarios without Global Human Footprint data; Figure S4: Changes in the distribution of *C. acuminata* under four scenarios from the current period to the 2050s and from the current period to the 2070s.
