# Supplementary material for: Insight into the Influence of Ecological Factors on Shaping Distribution Patterns of Camptotheca acuminata for Conservation and Management
Source: Plants (Basel). 2025 May 14;14(10):1466. doi: 10.3390/plants14101466 (PMC12114703; doi:10.3390/plants14101466)
Supplement: Supplementary file 1 [file plants-14-01466-s001.zip › plants-3561135-supplementary.pdf]

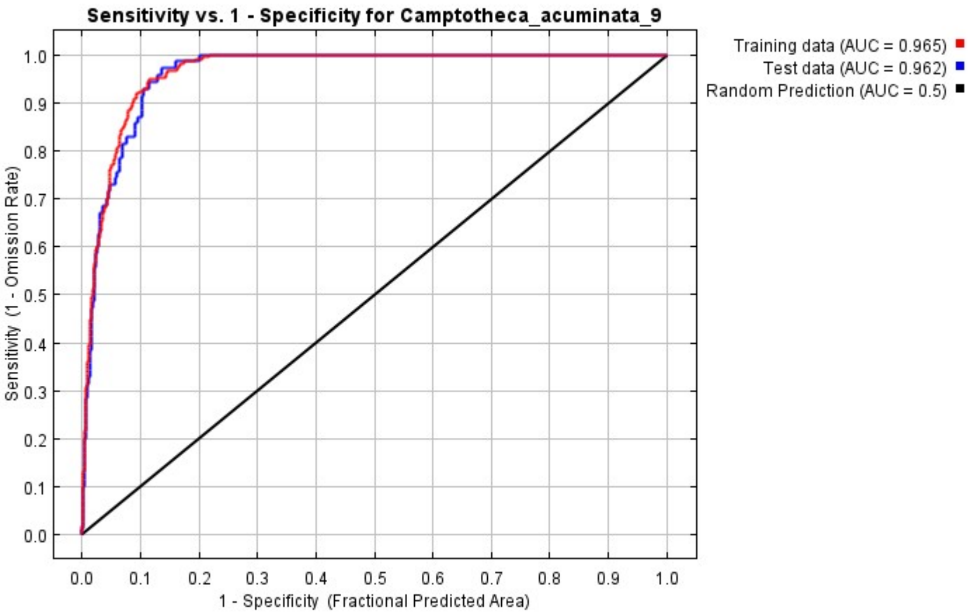

Figure S1. ROC curve of the MaxEnt model.

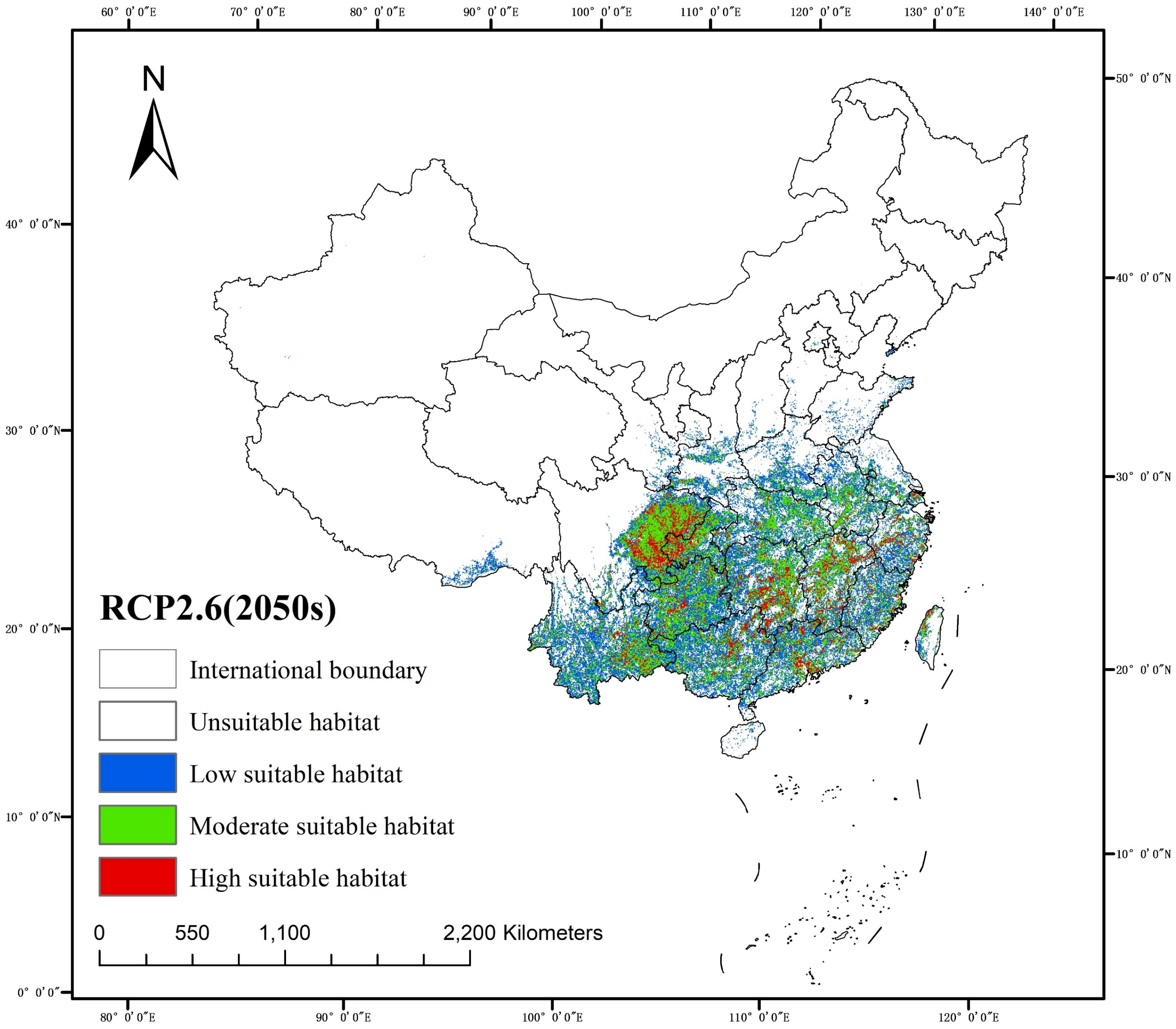
60° 0′ 0″E
70° 0′ 0″E
80° 0′ 0″E
90° 0′ 0″E
100° 0′ 0″E
110° 0′ 0″E
120° 0′ 0″E
130° 0′ 0″E
140° 0′ 0″E
N
40° 0′ 0″N
30° 0′ 0″N
20° 0′ 0″N
10° 0′ 0″N
0° 0′ 0″
50° 0′ 0″N
40° 0′ 0″N
30° 0′ 0″N
20° 0′ 0″N
10° 0′ 0″N
RCP2.6(2050s)
International boundary
Unsuitable habitat
Low suitable habitat
Moderate suitable habitat
High suitable habitat
0
550
1,100
2,200 Kilometers
80° 0′ 0″E
90° 0′ 0″E
100° 0′ 0″E
110° 0′ 0″E
120° 0′ 0″E

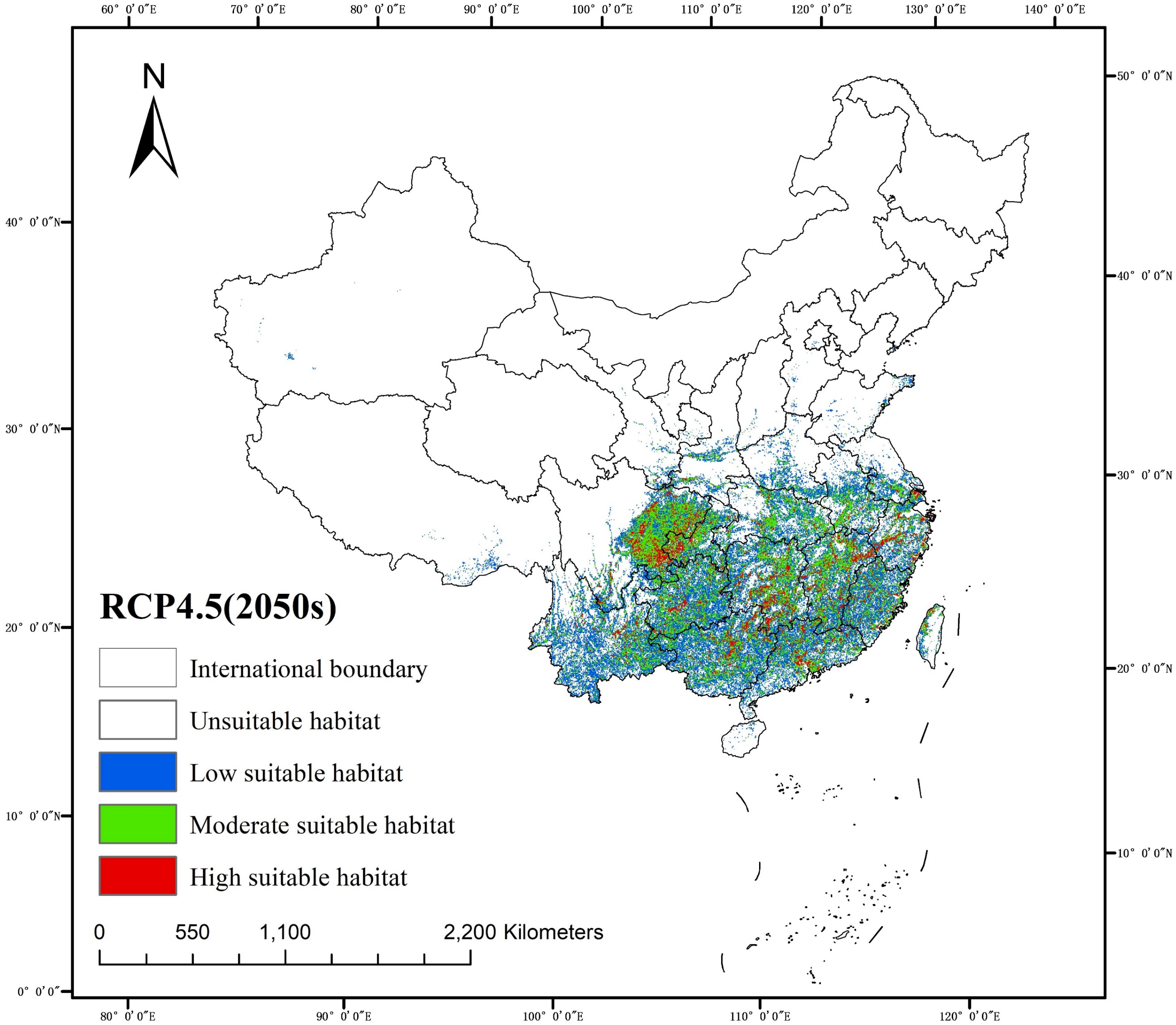
RCP4.5(2050s)
N
International boundary
Unsuitable habitat
Low suitable habitat
Moderate suitable habitat
High suitable habitat
0
550
1,100
2,200 Kilometers

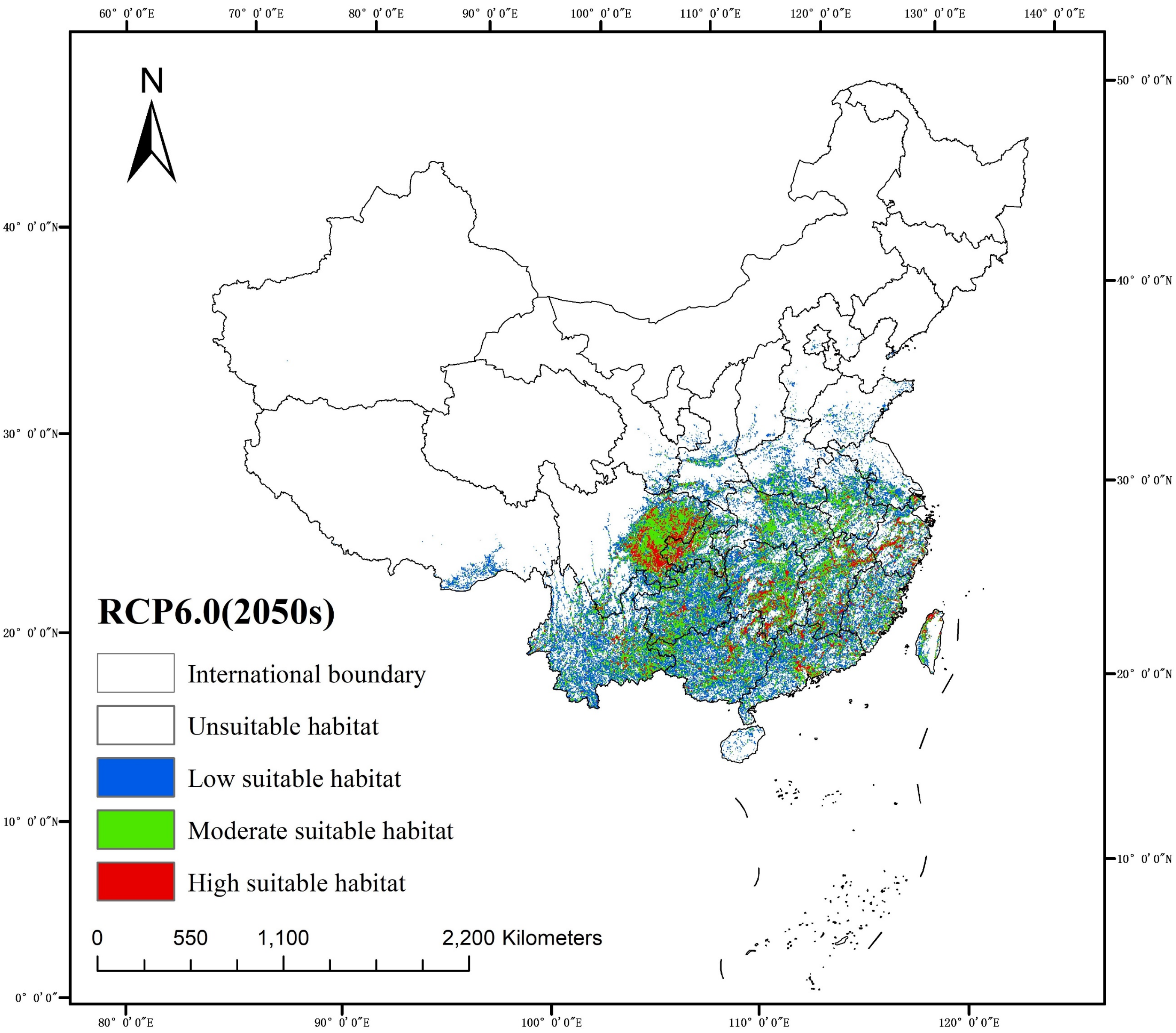
60° 0′0″E
70° 0′0″E
80° 0′0″E
90° 0′0″E
100° 0′0″E
110° 0′0″E
120° 0′0″E
130° 0′0″E
140° 0′0″E
50° 0′0″N
40° 0′0″N
30° 0′0″N
20° 0′0″N
10° 0′0″N
0° 0′0″
N
RCP6.0(2050s)
International boundary
Unsuitable habitat
Low suitable habitat
Moderate suitable habitat
High suitable habitat
0
550
1,100
2,200 Kilometers

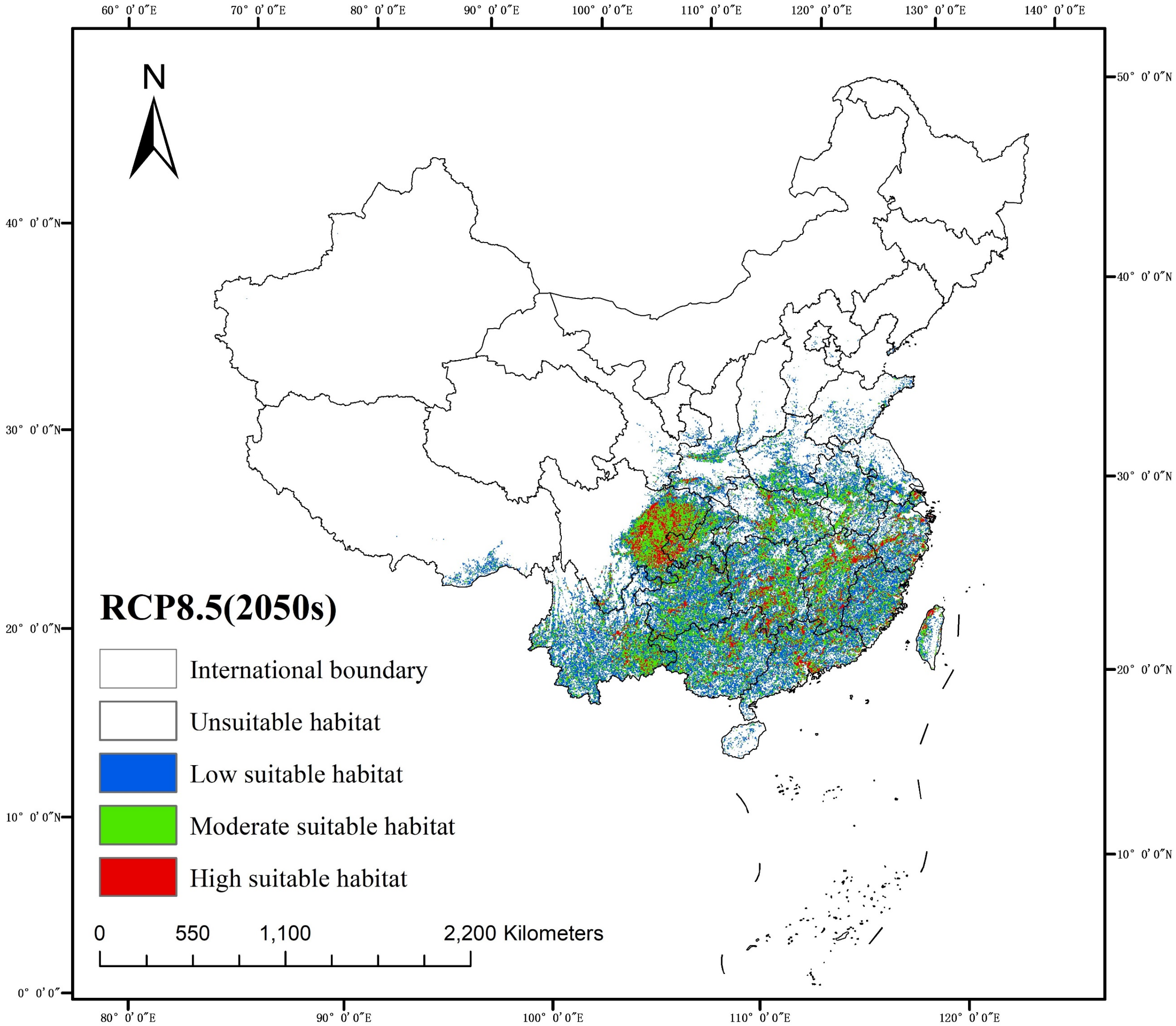

RCP8.5(2050s)
International boundary
Unsuitable habitat
Low suitable habitat
Moderate suitable habitat
High suitable habitat
0
550
1,100
2,200 Kilometers
N

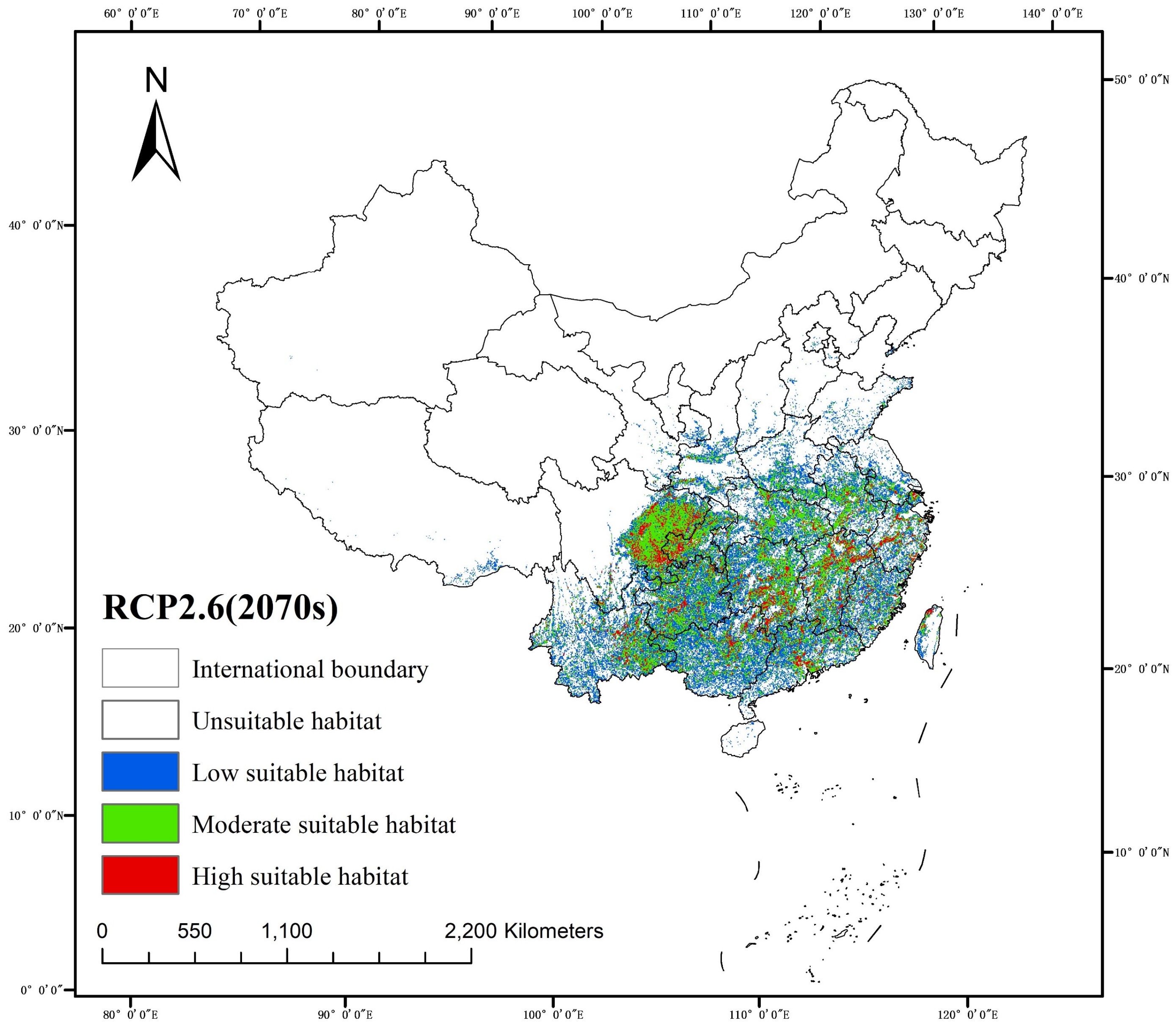

60° 0′ 0″E
70° 0′ 0″E
80° 0′ 0″E
90° 0′ 0″E
100° 0′ 0″E
110° 0′ 0″E
120° 0′ 0″E
130° 0′ 0″E
140° 0′ 0″E
50° 0′ 0″N
40° 0′ 0″N
30° 0′ 0″N
20° 0′ 0″N
10° 0′ 0″N
0° 0′ 0″
N
RCP2.6(2070s)
International boundary
Unsuitable habitat
Low suitable habitat
Moderate suitable habitat
High suitable habitat
0
550
1,100
2,200 Kilometers
80° 0′ 0″E
90° 0′ 0″E
100° 0′ 0″E
110° 0′ 0″E
120° 0′ 0″E

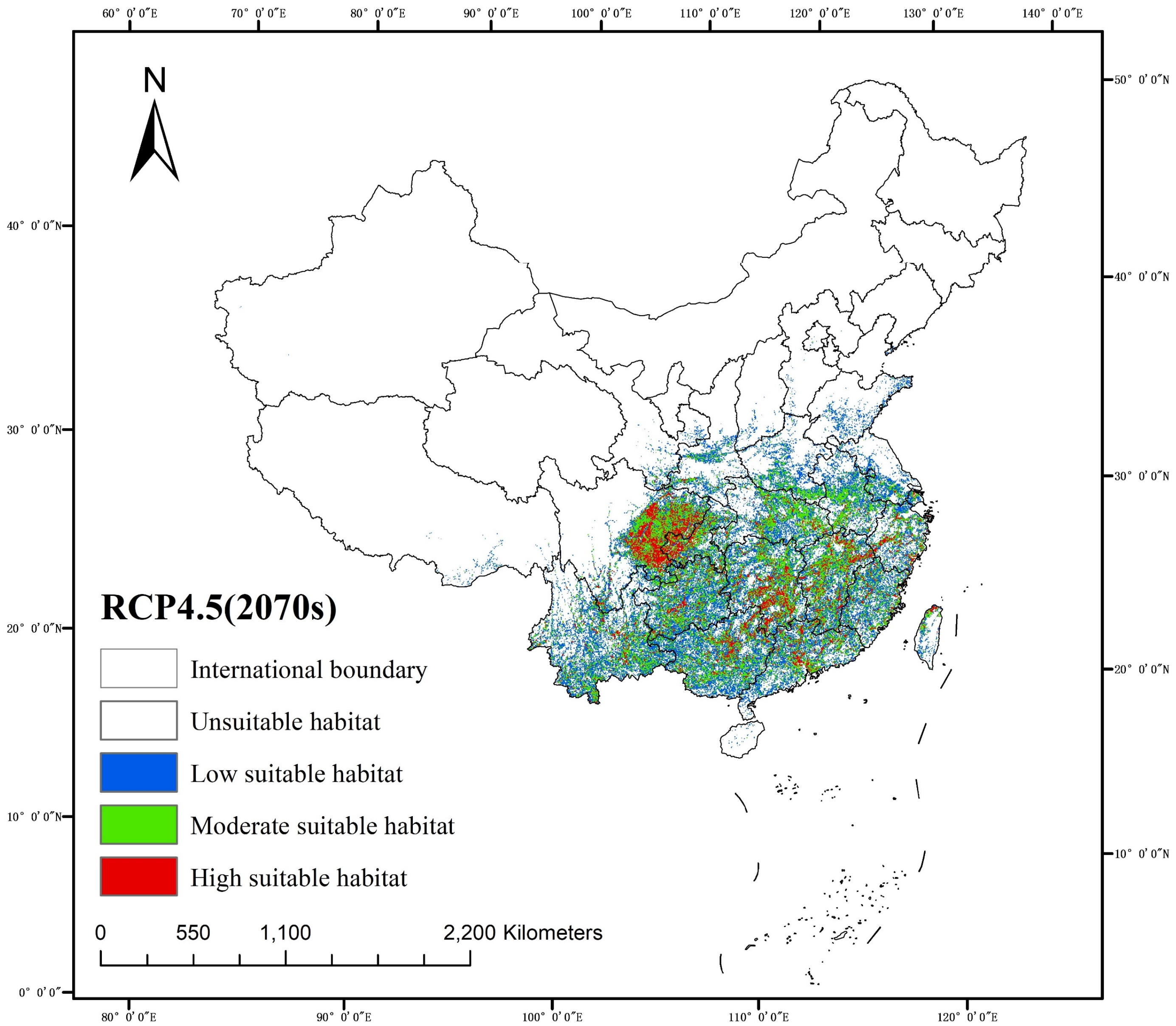
60° 0′ 0″E
70° 0′ 0″E
80° 0′ 0″E
90° 0′ 0″E
100° 0′ 0″E
110° 0′ 0″E
120° 0′ 0″E
130° 0′ 0″E
140° 0′ 0″E
N
50° 0′ 0″N
40° 0′ 0″N
30° 0′ 0″N
20° 0′ 0″N
10° 0′ 0″N
0° 0′ 0″
RCP4.5(2070s)
International boundary
Unsuitable habitat
Low suitable habitat
Moderate suitable habitat
High suitable habitat
0
550
1,100
2,200 Kilometers

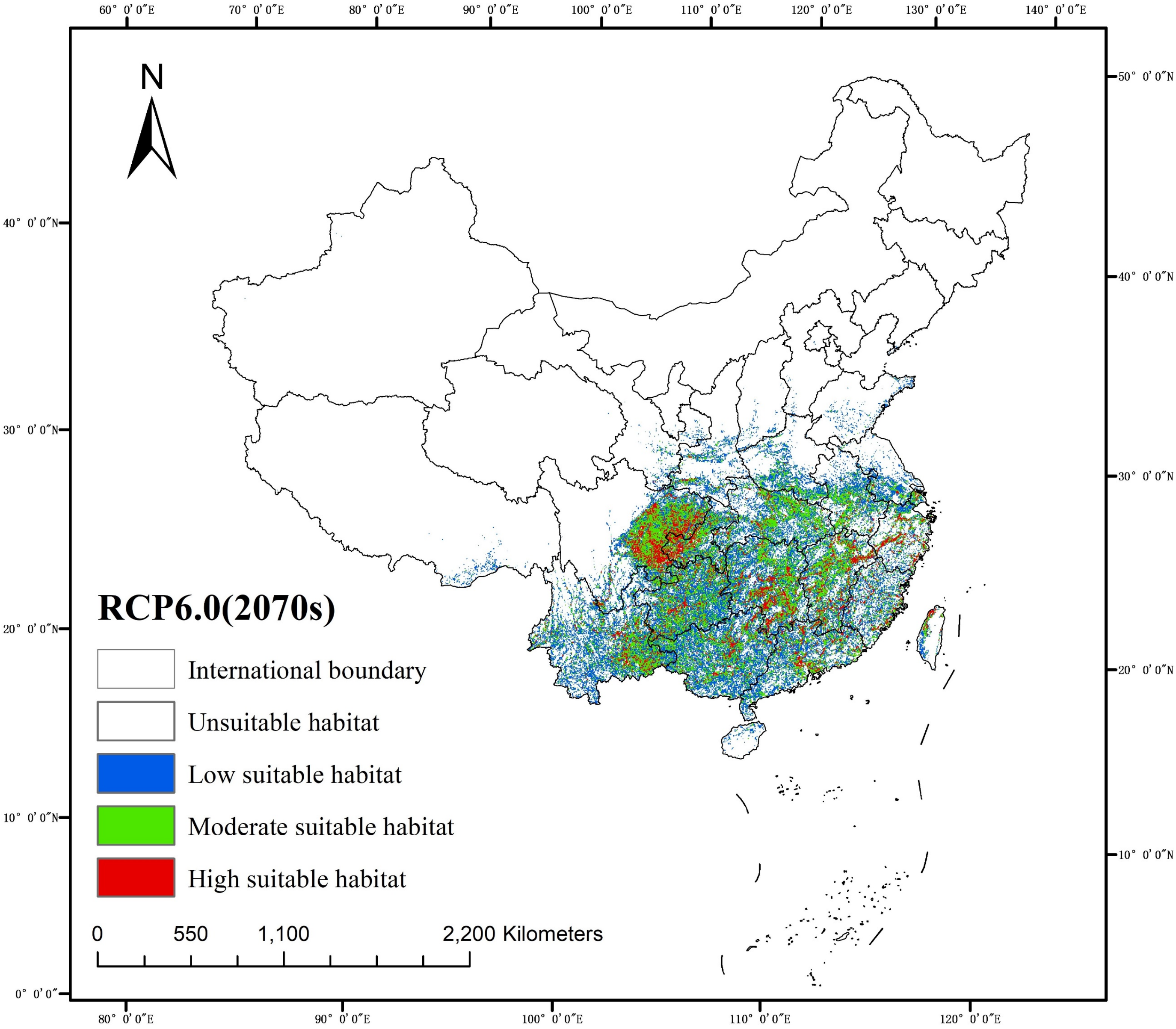

RCP6.0(2070s)
International boundary
Unsuitable habitat
Low suitable habitat
Moderate suitable habitat
High suitable habitat
0
550
1,100
2,200 Kilometers
N
60° 0′ 0″E
70° 0′ 0″E
80° 0′ 0″E
90° 0′ 0″E
100° 0′ 0″E
110° 0′ 0″E
120° 0′ 0″E
130° 0′ 0″E
140° 0′ 0″E
50° 0′ 0″N
40° 0′ 0″N
30° 0′ 0″N
20° 0′ 0″N
10° 0′ 0″N
0° 0′ 0″

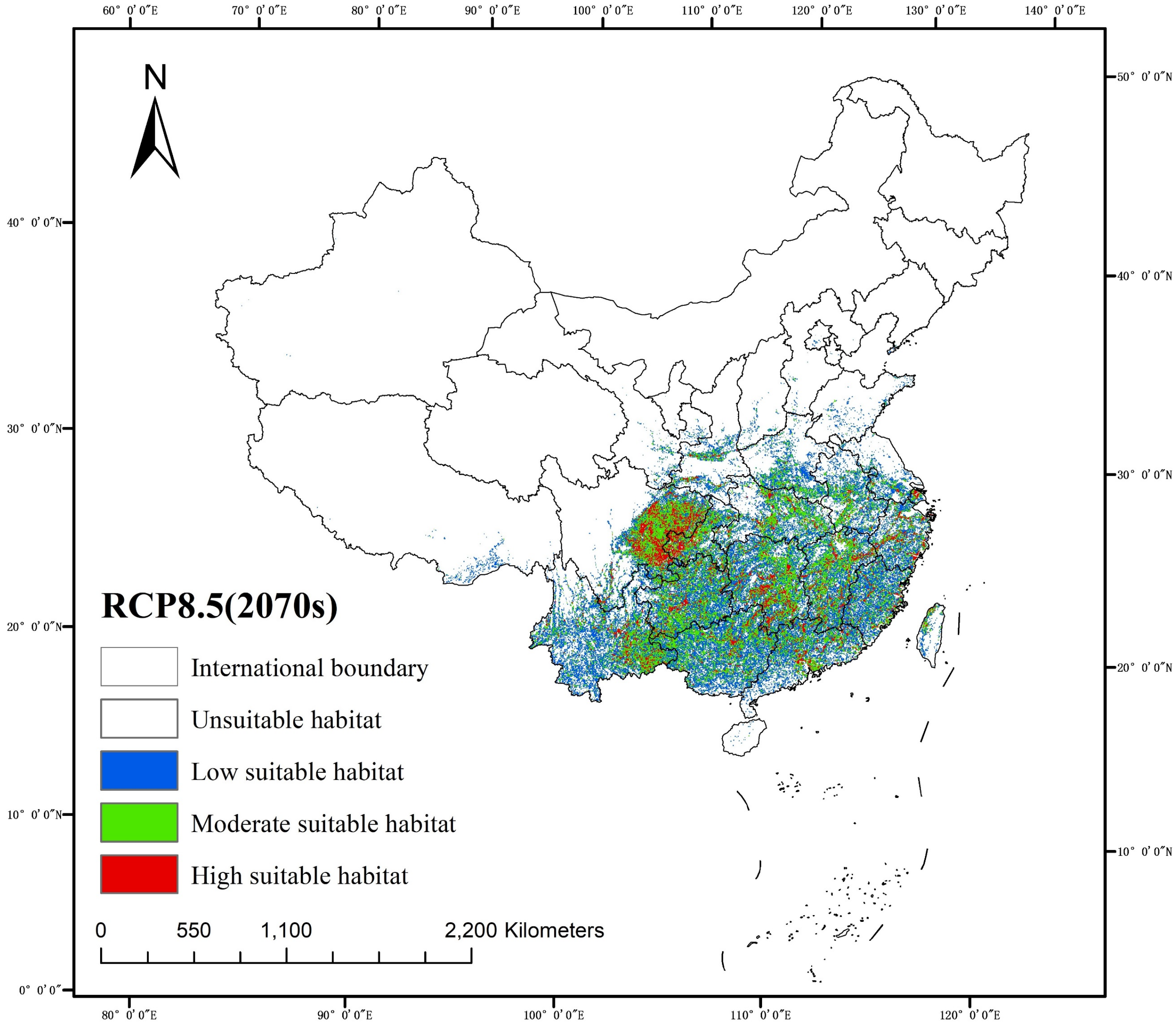

Figure S2. Distribution of *C.acuminata* in the 2050s and 2070s under different greenhouse gases concentration scenarios.

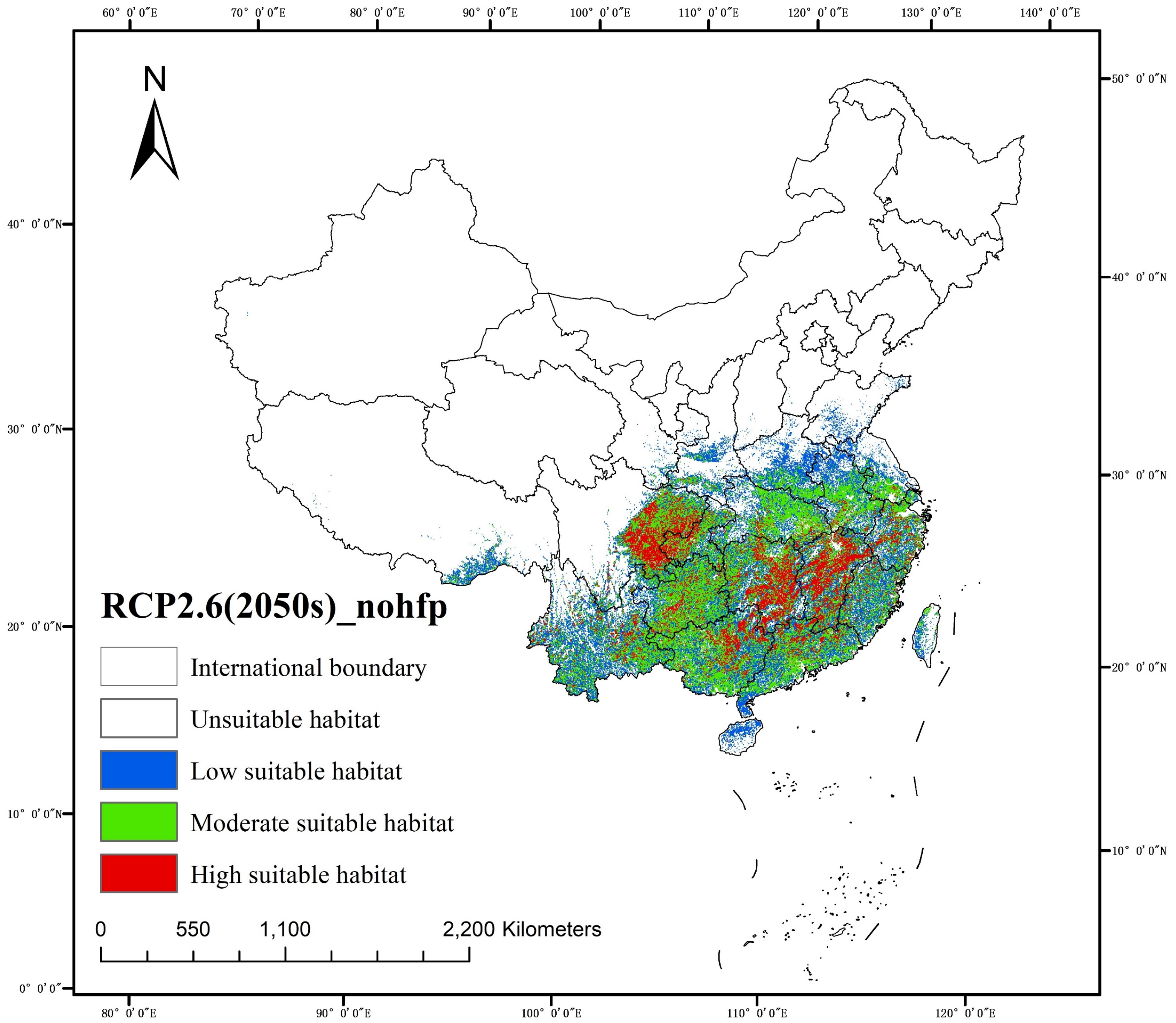
RCP2.6(2050s)_nohfp
International boundary
Unsuitable habitat
Low suitable habitat
Moderate suitable habitat
High suitable habitat
0 550 1,100 2,200 Kilometers
N
60° 0′0″E
70° 0′0″E
80° 0′0″E
90° 0′0″E
100° 0′0″E
110° 0′0″E
120° 0′0″E
130° 0′0″E
140° 0′0″E
50° 0′0″N
40° 0′0″N
30° 0′0″N
20° 0′0″N
10° 0′0″N
0° 0′0″

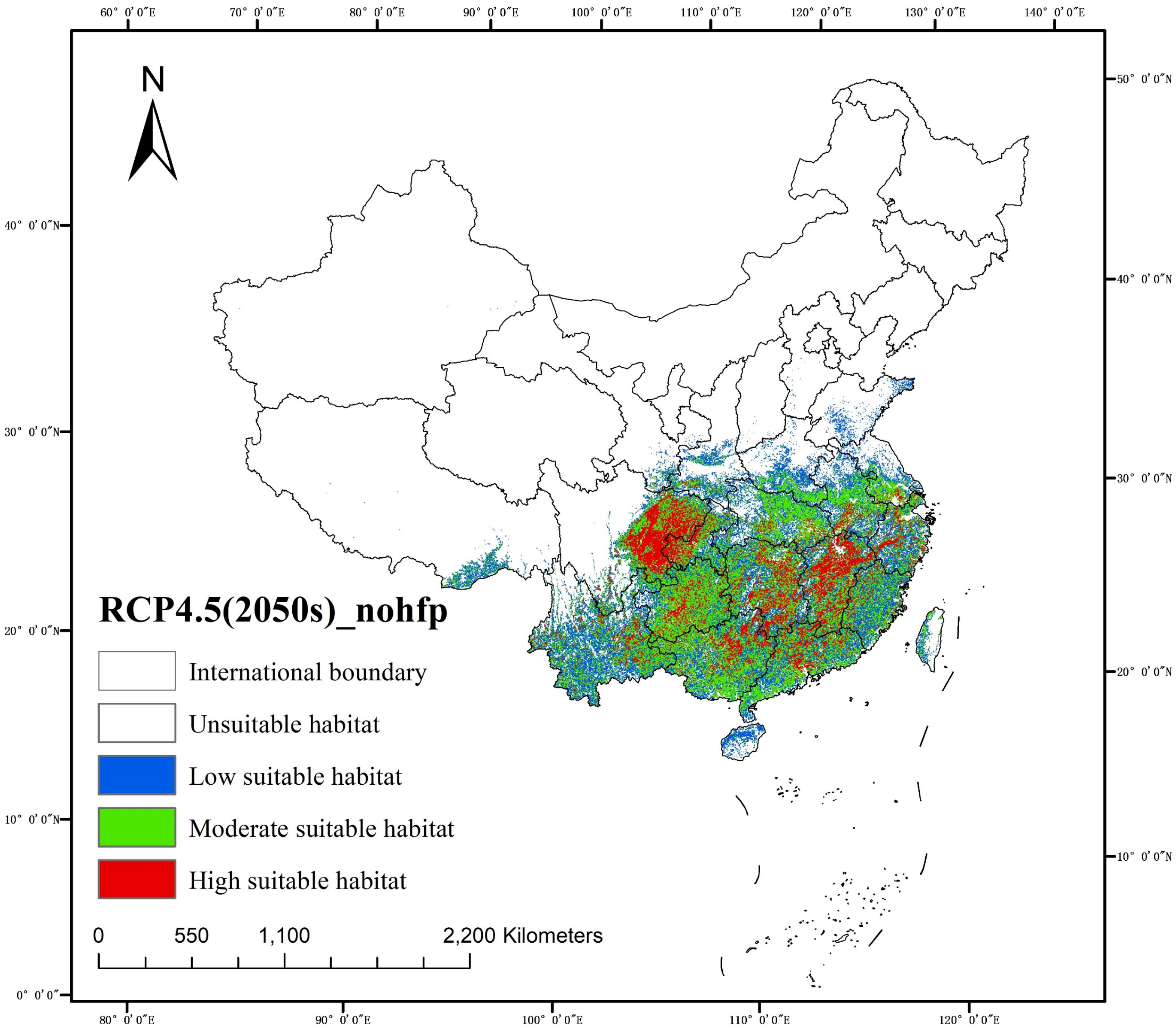
RCP4.5(2050s)_nohfp
International boundary
Unsuitable habitat
Low suitable habitat
Moderate suitable habitat
High suitable habitat
0
550
1,100
2,200 Kilometers
N
60° 0′ 0″E
70° 0′ 0″E
80° 0′ 0″E
90° 0′ 0″E
100° 0′ 0″E
110° 0′ 0″E
120° 0′ 0″E
130° 0′ 0″E
140° 0′ 0″E
50° 0′ 0″N
40° 0′ 0″N
30° 0′ 0″N
20° 0′ 0″N
10° 0′ 0″N
0° 0′ 0″

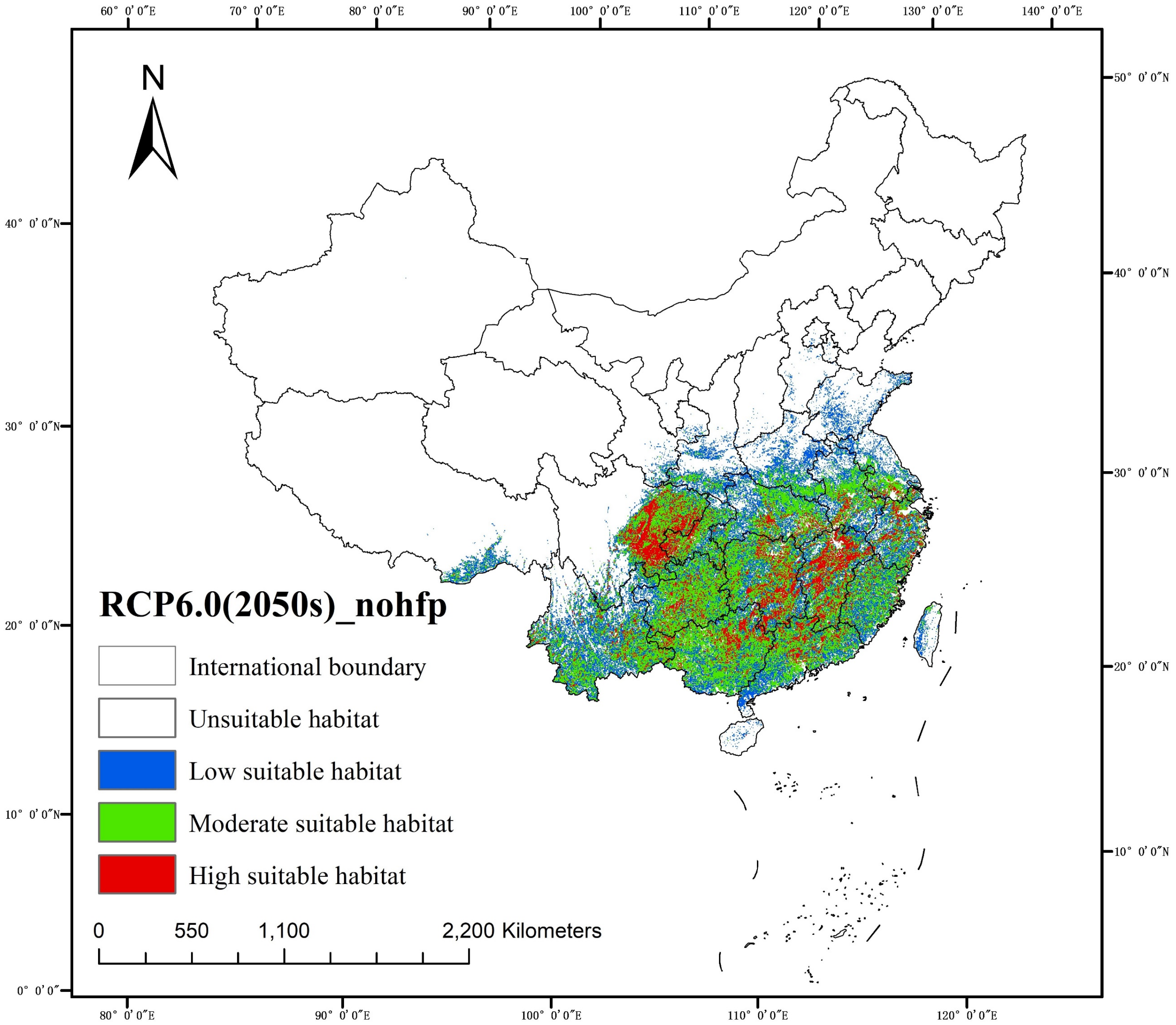
60° 0′ 0″E
70° 0′ 0″E
80° 0′ 0″E
90° 0′ 0″E
100° 0′ 0″E
110° 0′ 0″E
120° 0′ 0″E
130° 0′ 0″E
140° 0′ 0″E
50° 0′ 0″N
40° 0′ 0″N
30° 0′ 0″N
20° 0′ 0″N
10° 0′ 0″N
0° 0′ 0″
N
RCP6.0(2050s)_nohfp
International boundary
Unsuitable habitat
Low suitable habitat
Moderate suitable habitat
High suitable habitat
0
550
1,100
2,200 Kilometers

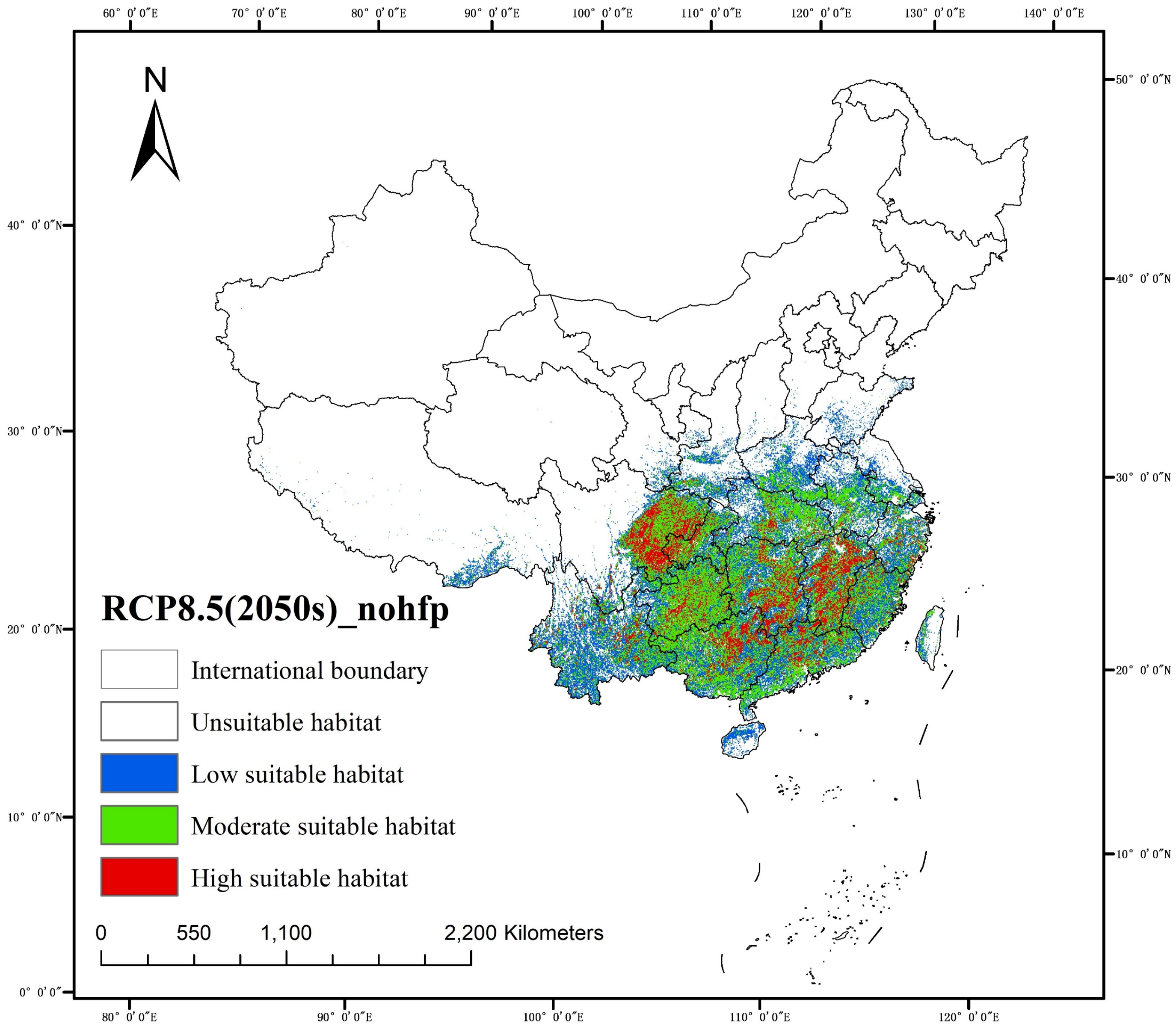

RCP8.5(2050s)_nohfp
International boundary
Unsuitable habitat
Low suitable habitat
Moderate suitable habitat
High suitable habitat
0 550 1,100 2,200 Kilometers
N

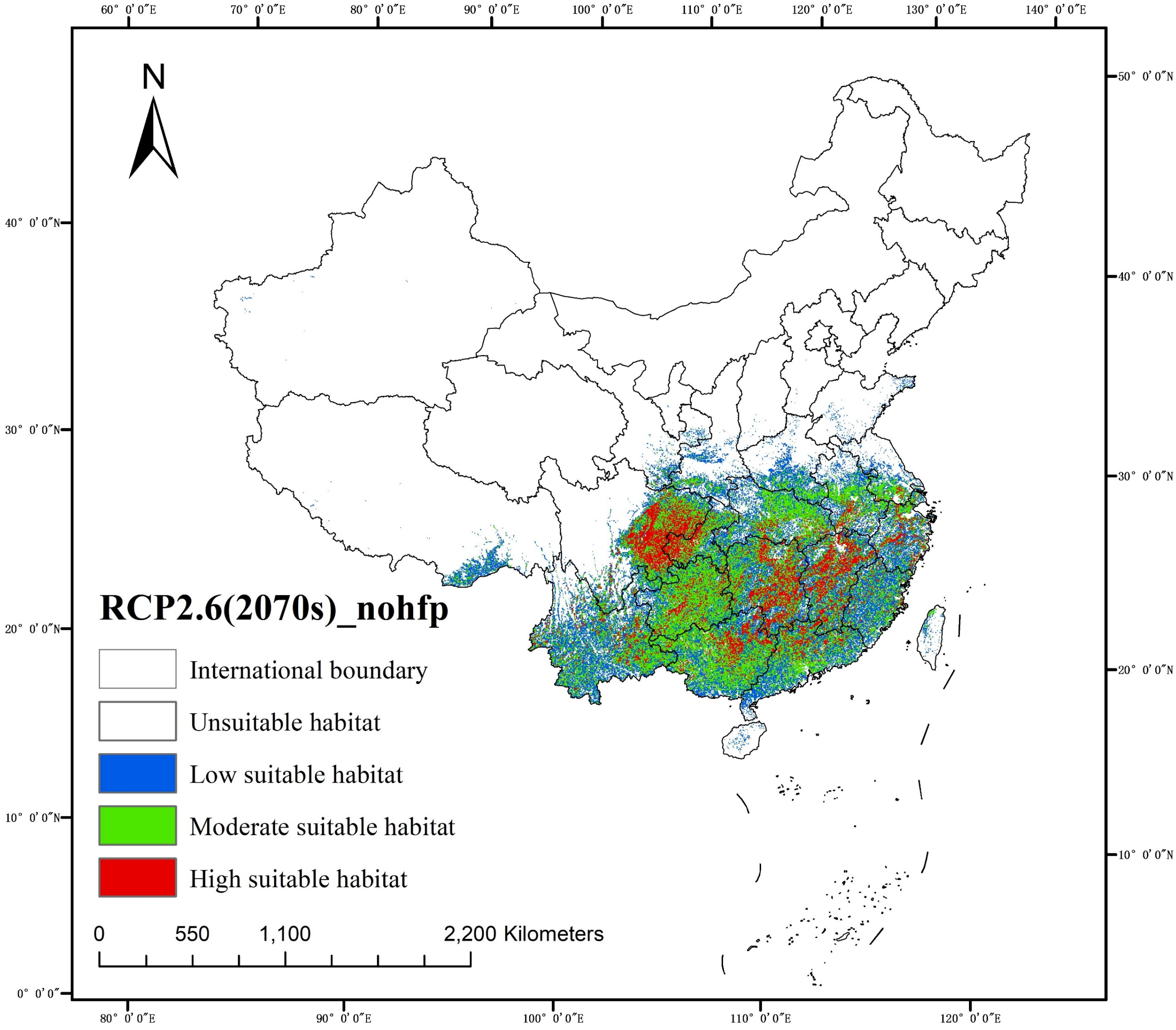

N
RCP2.6(2070s)_nohfp
International boundary
Unsuitable habitat
Low suitable habitat
Moderate suitable habitat
High suitable habitat
0
550
1,100
2,200 Kilometers
60° 0′ 0″E
70° 0′ 0″E
80° 0′ 0″E
90° 0′ 0″E
100° 0′ 0″E
110° 0′ 0″E
120° 0′ 0″E
130° 0′ 0″E
140° 0′ 0″E
50° 0′ 0″N
40° 0′ 0″N
30° 0′ 0″N
20° 0′ 0″N
10° 0′ 0″N
0° 0′ 0″

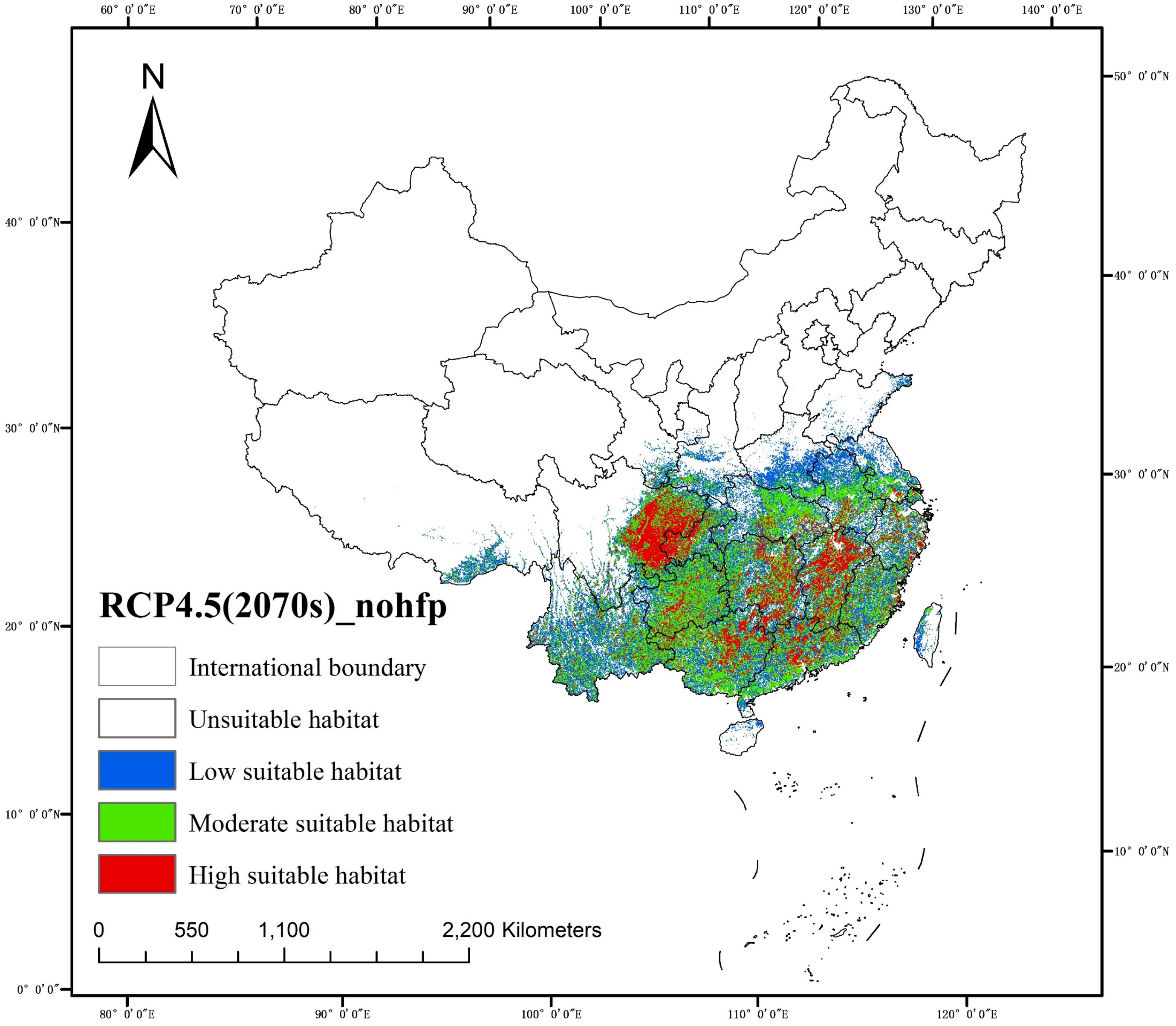

RCP4.5(2070s)_nohfp
International boundary
Unsuitable habitat
Low suitable habitat
Moderate suitable habitat
High suitable habitat
0 550 1,100 2,200 Kilometers
N

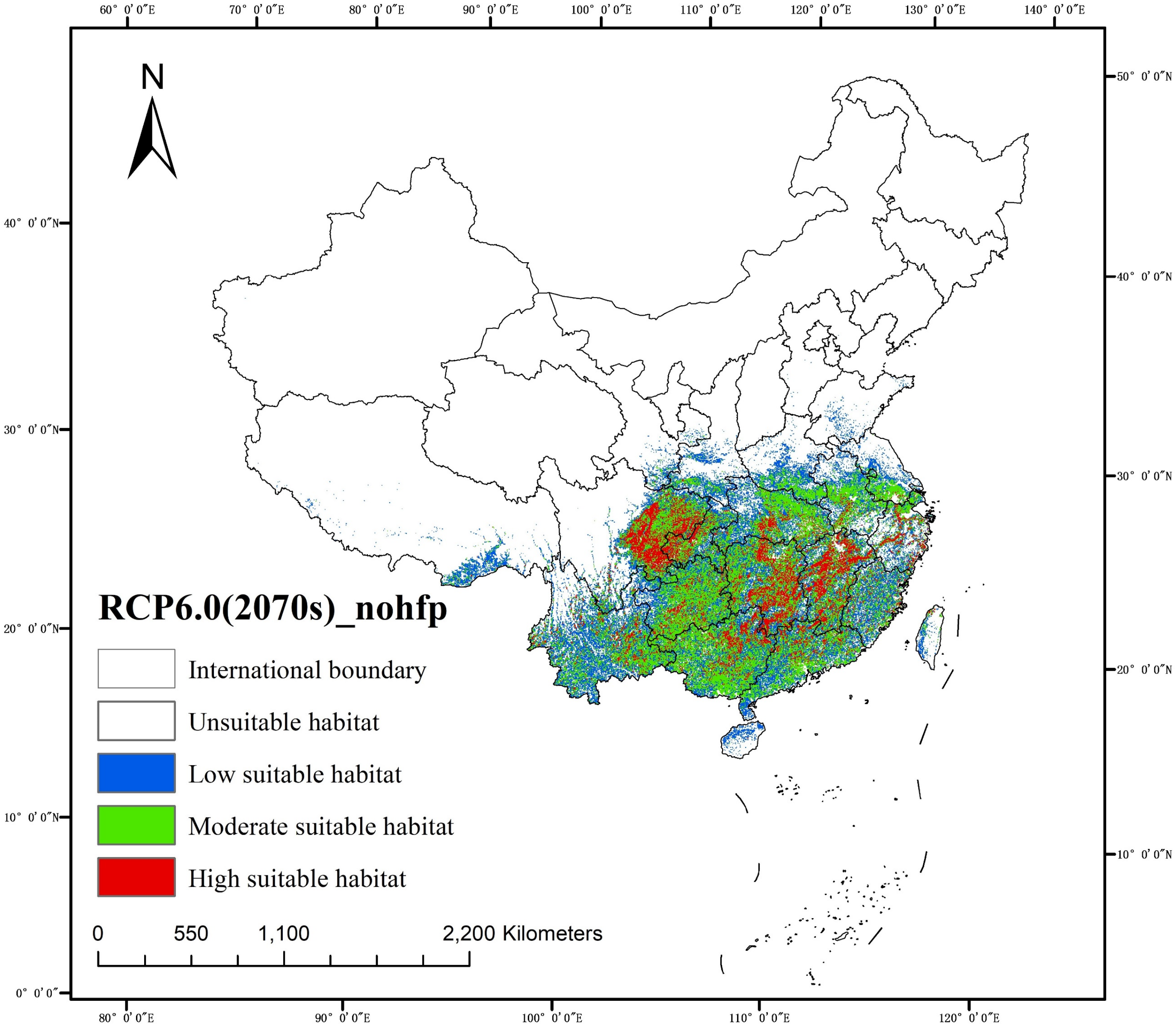

N
RCP6.0(2070s)_nohfp
International boundary
Unsuitable habitat
Low suitable habitat
Moderate suitable habitat
High suitable habitat
0 550 1,100 2,200 Kilometers
60° 0′0″E
70° 0′0″E
80° 0′0″E
90° 0′0″E
100° 0′0″E
110° 0′0″E
120° 0′0″E
130° 0′0″E
140° 0′0″E
50° 0′0″N
40° 0′0″N
30° 0′0″N
20° 0′0″N
10° 0′0″N
0° 0′0″

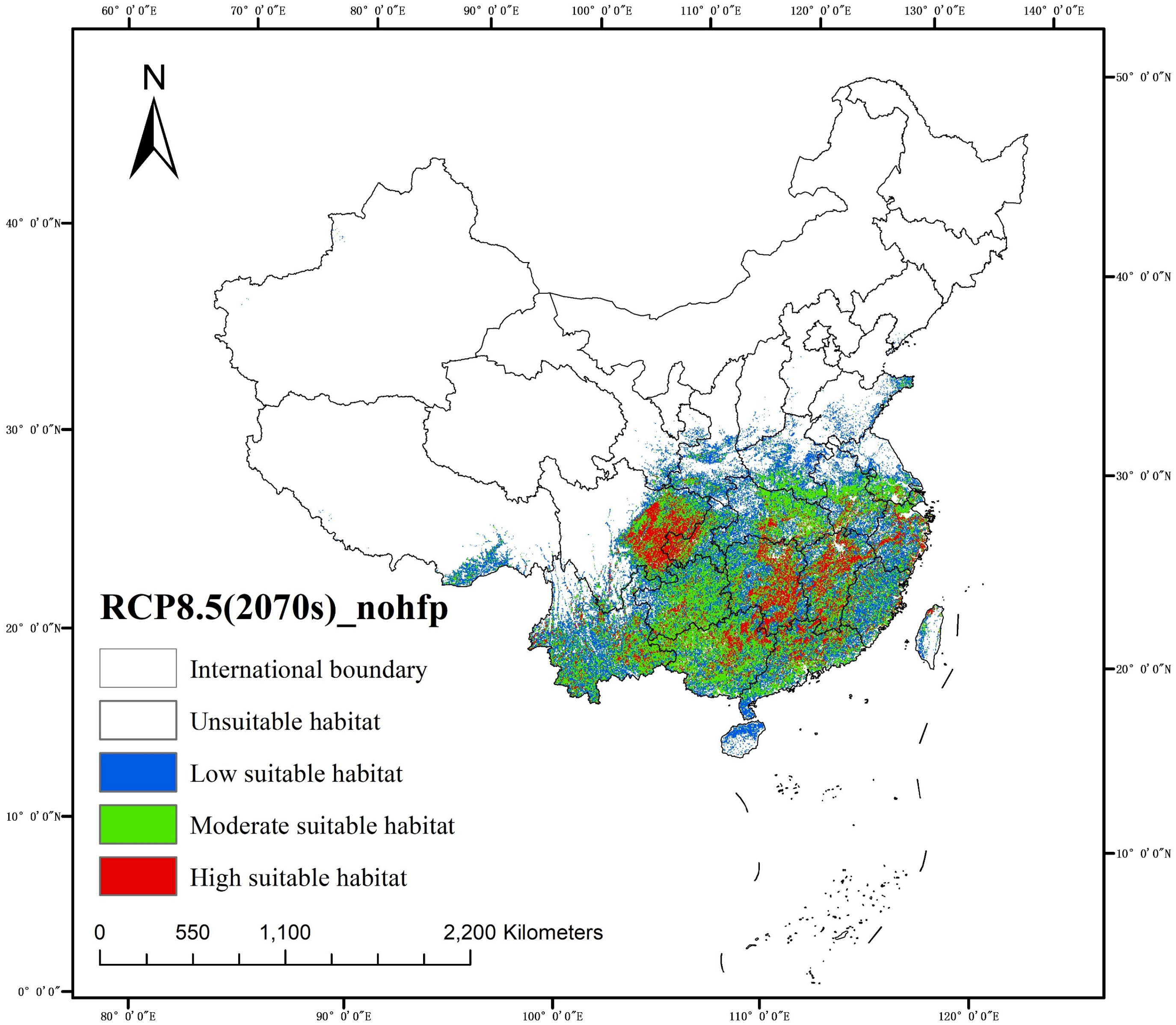

Figure S 3. Distribution of *C.acuminata* in the 2050s and 2070s under different greenhouse gases concentration scenarios without global human footprint.

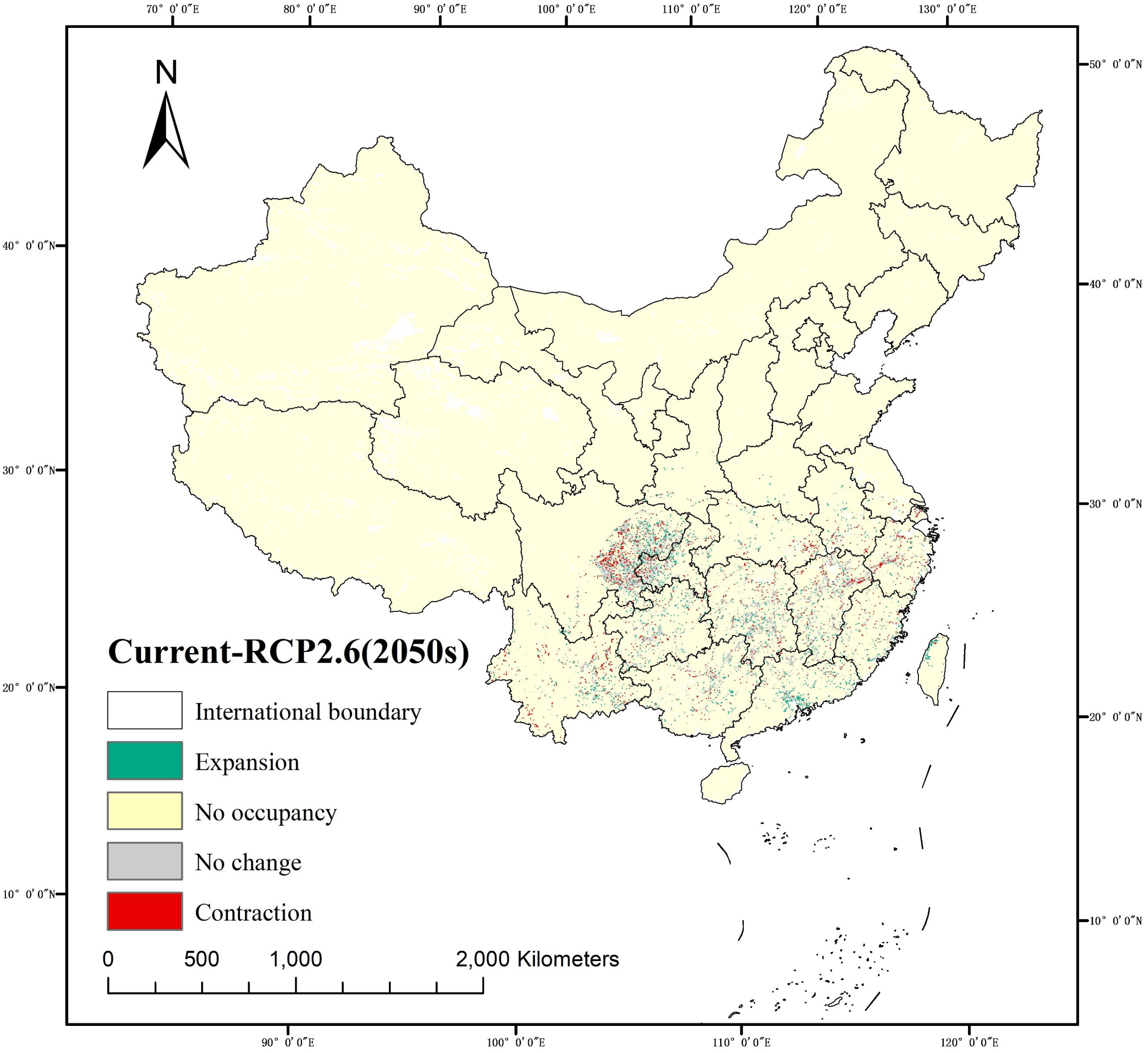

70° 0′ 0″E
80° 0′ 0″E
90° 0′ 0″E
100° 0′ 0″E
110° 0′ 0″E
120° 0′ 0″E
130° 0′ 0″E
50° 0′ 0″N
40° 0′ 0″N
30° 0′ 0″N
20° 0′ 0″N
10° 0′ 0″N
N
Current-RCP2.6(2050s)
International boundary
Expansion
No occupancy
No change
Contraction
0
500
1,000
2,000 Kilometers

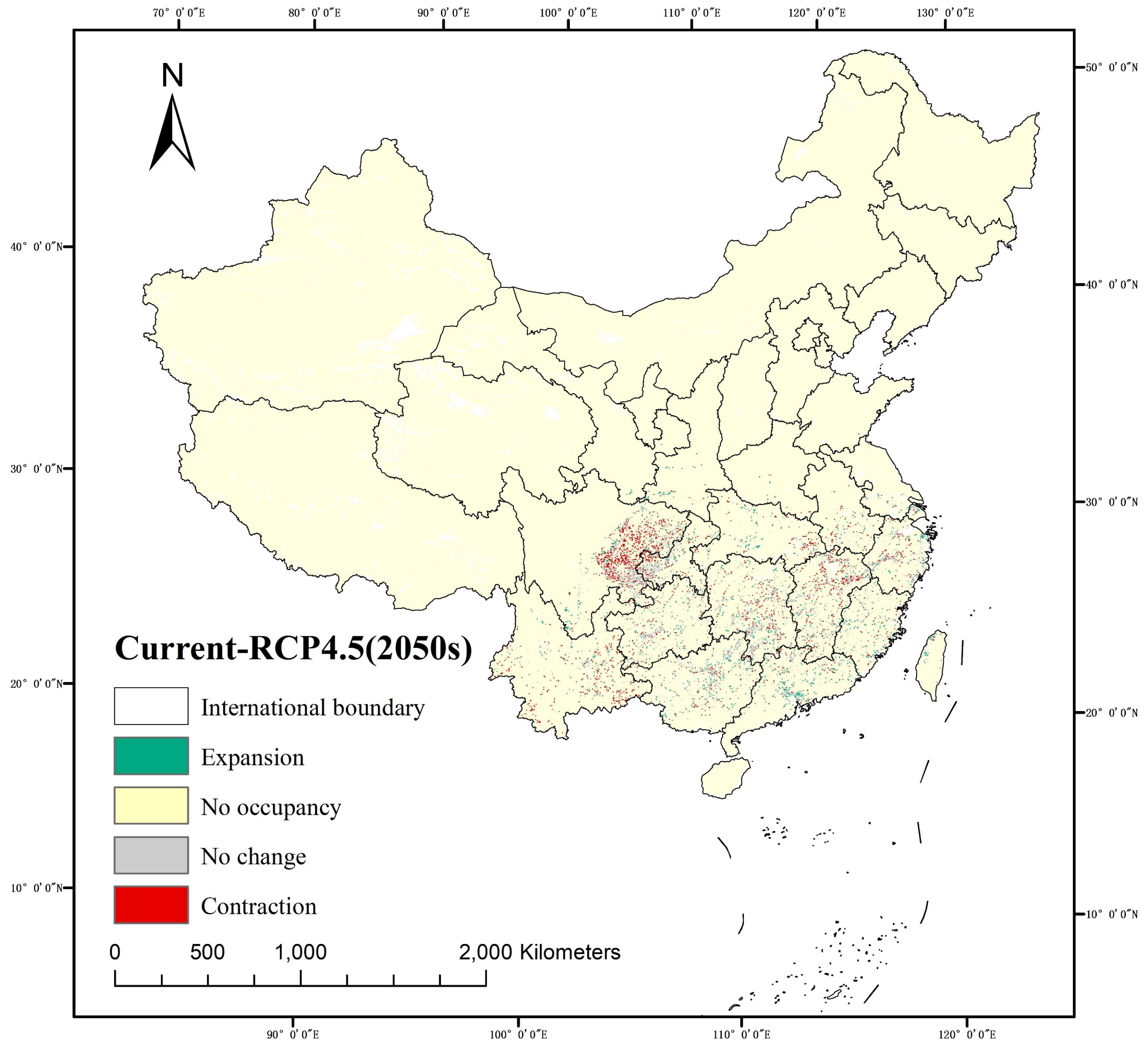

70° 0′ 0″E
80° 0′ 0″E
90° 0′ 0″E
100° 0′ 0″E
110° 0′ 0″E
120° 0′ 0″E
130° 0′ 0″E
N
40° 0′ 0″N
30° 0′ 0″N
20° 0′ 0″N
10° 0′ 0″N
50° 0′ 0″N
Current-RCP4.5(2050s)
International boundary
Expansion
No occupancy
No change
Contraction
0
500
1,000
2,000 Kilometers

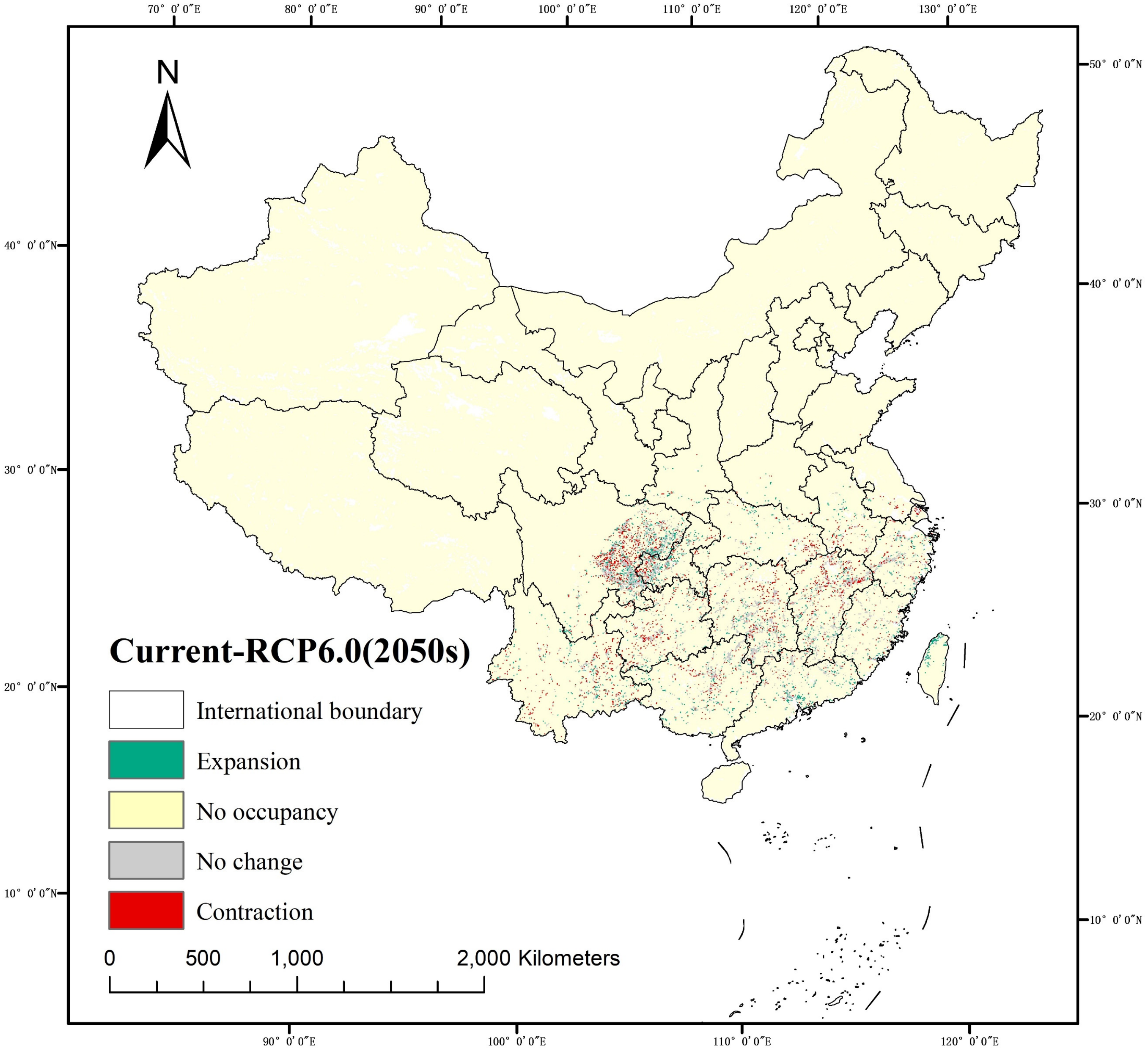
N
Current-RCP6.0(2050s)
International boundary
Expansion
No occupancy
No change
Contraction
0
500
1,000
2,000 Kilometers
70° 0′ 0″E
80° 0′ 0″E
90° 0′ 0″E
100° 0′ 0″E
110° 0′ 0″E
120° 0′ 0″E
130° 0′ 0″E
50° 0′ 0″N
40° 0′ 0″N
30° 0′ 0″N
20° 0′ 0″N
10° 0′ 0″N

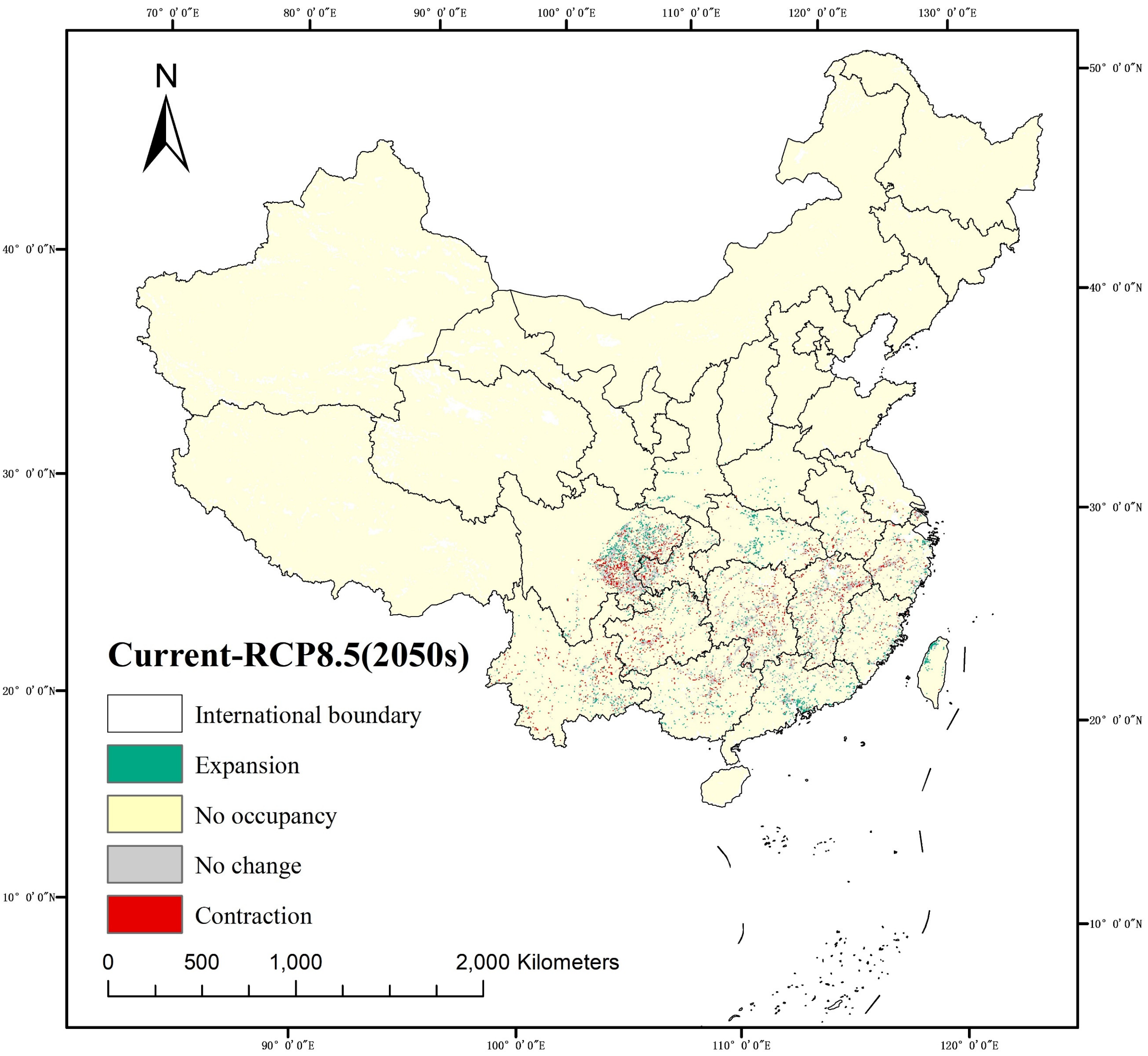

70° 0′ 0″E
80° 0′ 0″E
90° 0′ 0″E
100° 0′ 0″E
110° 0′ 0″E
120° 0′ 0″E
130° 0′ 0″E
50° 0′ 0″N
40° 0′ 0″N
30° 0′ 0″N
20° 0′ 0″N
10° 0′ 0″N
N
Current-RCP8.5(2050s)
International boundary
Expansion
No occupancy
No change
Contraction
0
500
1,000
2,000 Kilometers

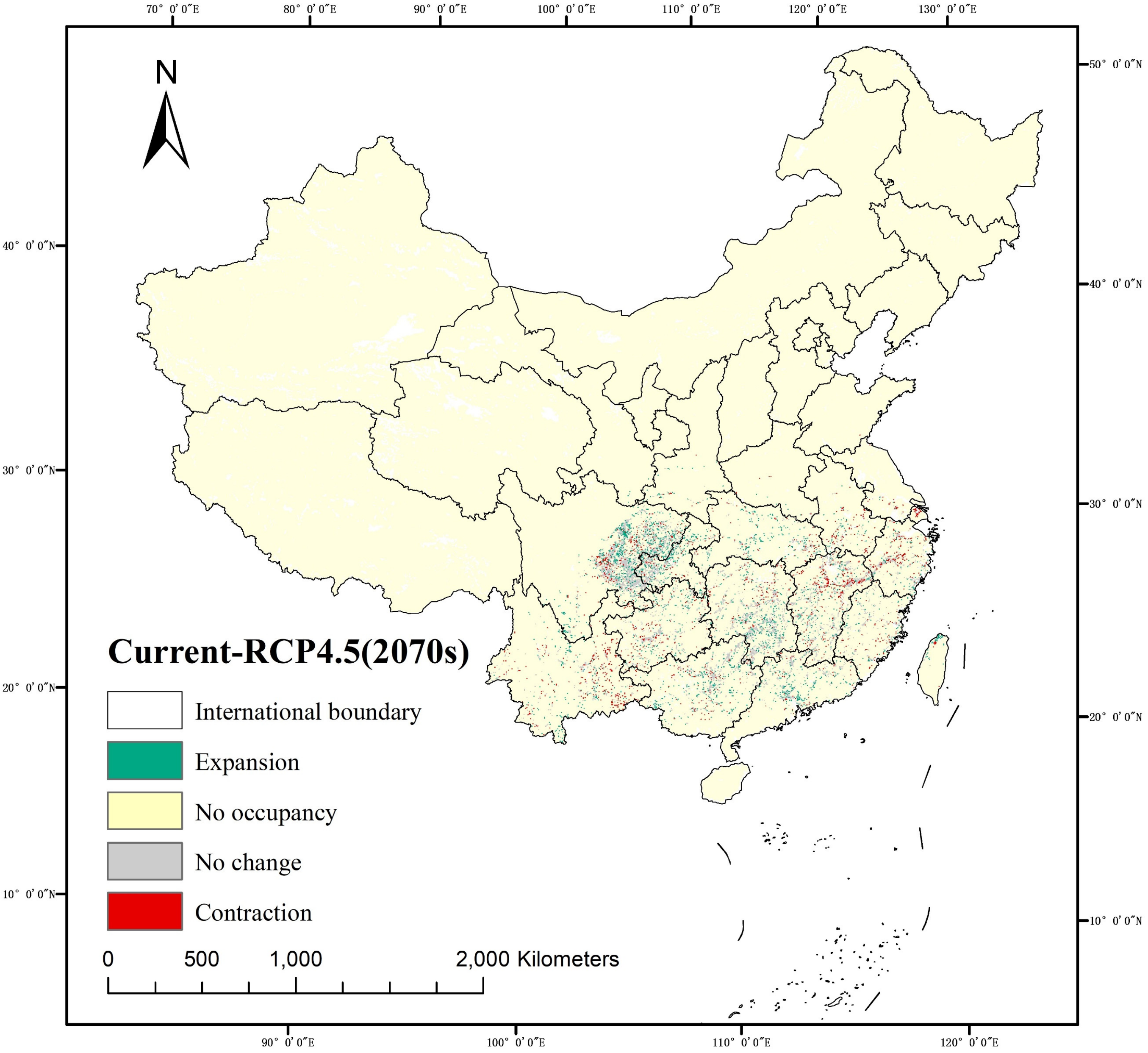
70° 0′ 0″E
80° 0′ 0″E
90° 0′ 0″E
100° 0′ 0″E
110° 0′ 0″E
120° 0′ 0″E
130° 0′ 0″E
50° 0′ 0″N
40° 0′ 0″N
30° 0′ 0″N
20° 0′ 0″N
10° 0′ 0″N
N
Current-RCP4.5(2070s)
International boundary
Expansion
No occupancy
No change
Contraction
0
500
1,000
2,000 Kilometers

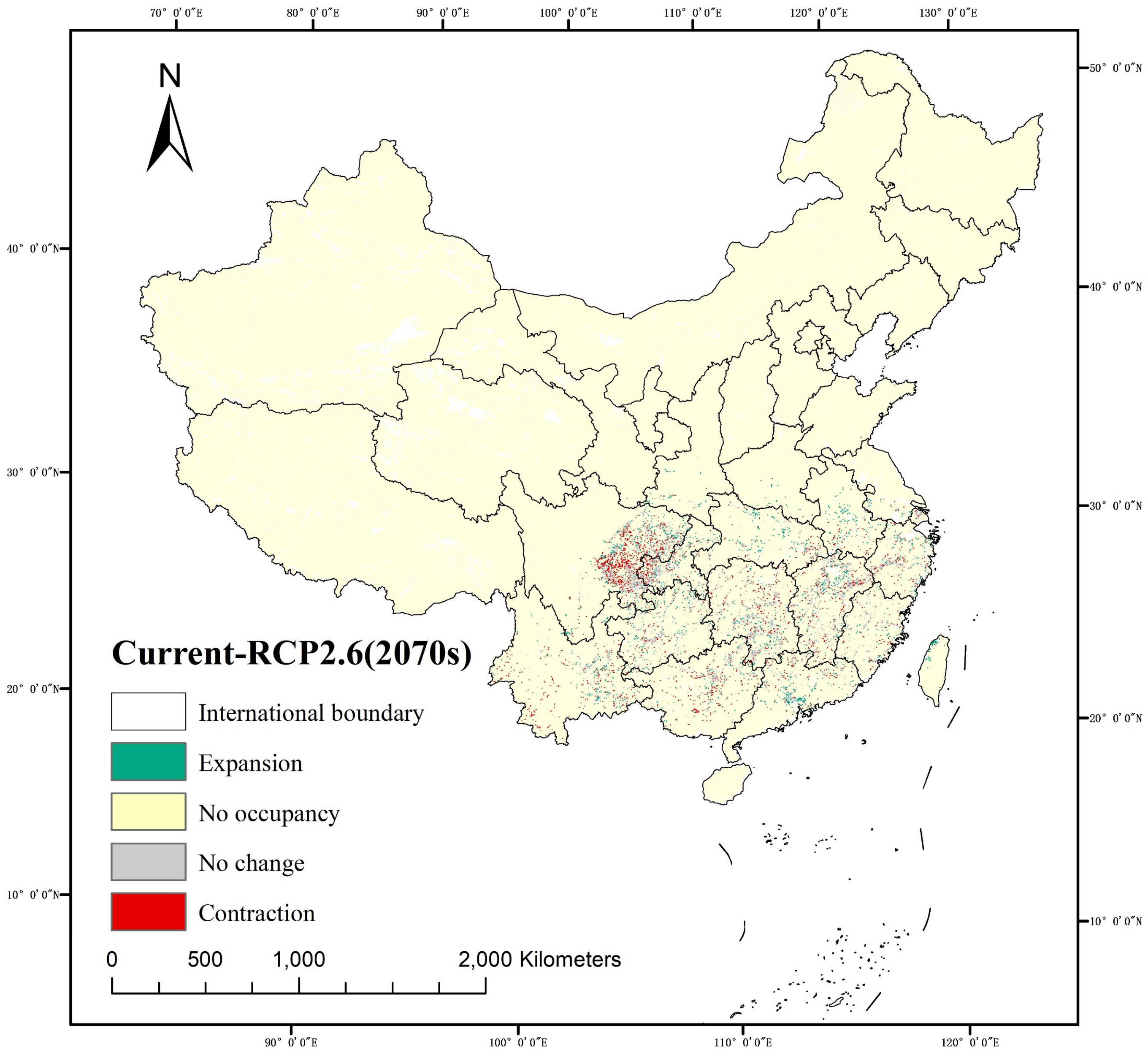

Current-RCP2.6(2070s)
International boundary
Expansion
No occupancy
No change
Contraction
0 500 1,000 2,000 Kilometers
N

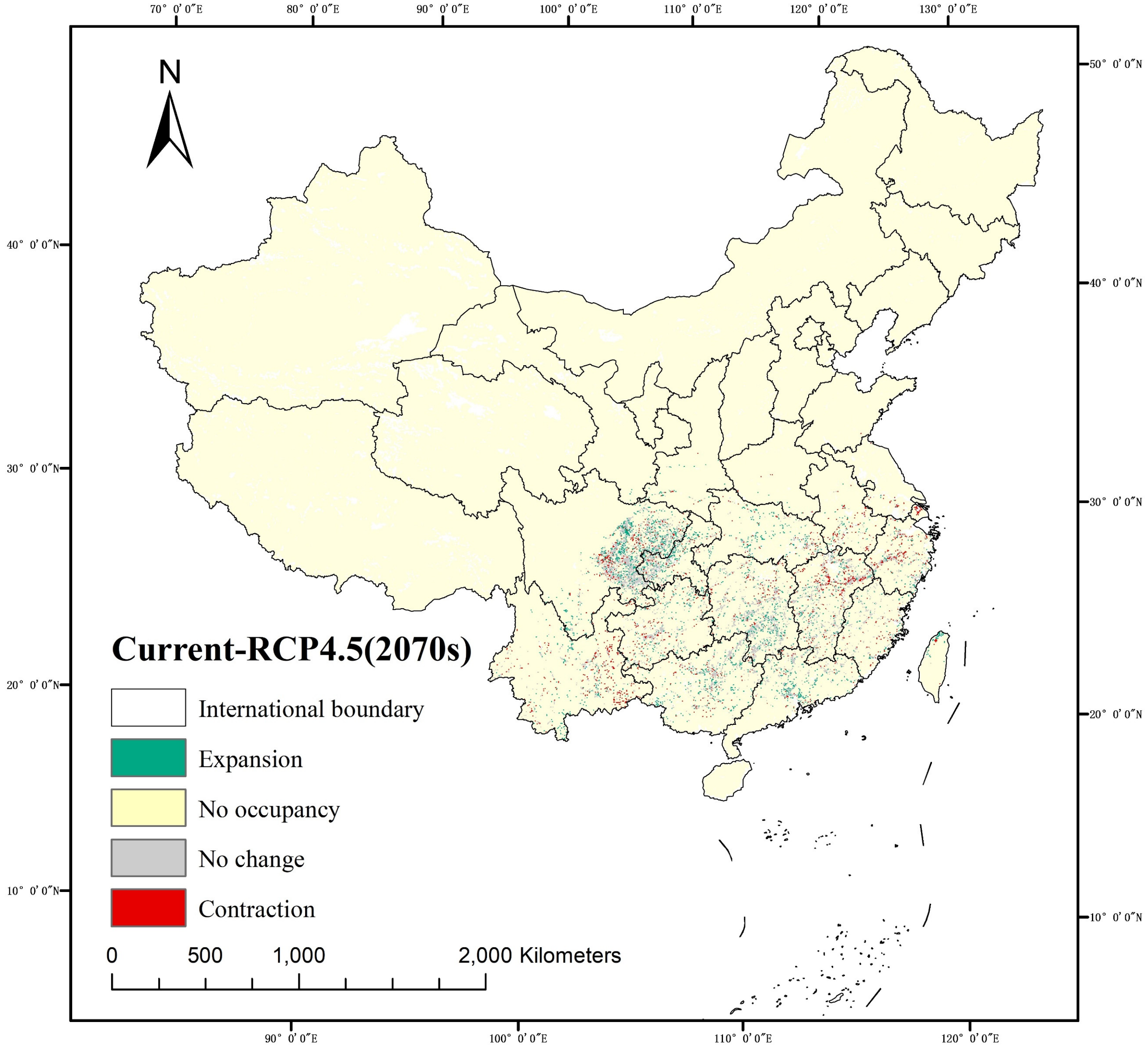
70° 0′ 0″E
80° 0′ 0″E
90° 0′ 0″E
100° 0′ 0″E
110° 0′ 0″E
120° 0′ 0″E
130° 0′ 0″E
50° 0′ 0″N
40° 0′ 0″N
30° 0′ 0″N
20° 0′ 0″N
10° 0′ 0″N
N
Current-RCP4.5(2070s)
International boundary
Expansion
No occupancy
No change
Contraction
0
500
1,000
2,000 Kilometers
90° 0′ 0″E
100° 0′ 0″E
110° 0′ 0″E
120° 0′ 0″E

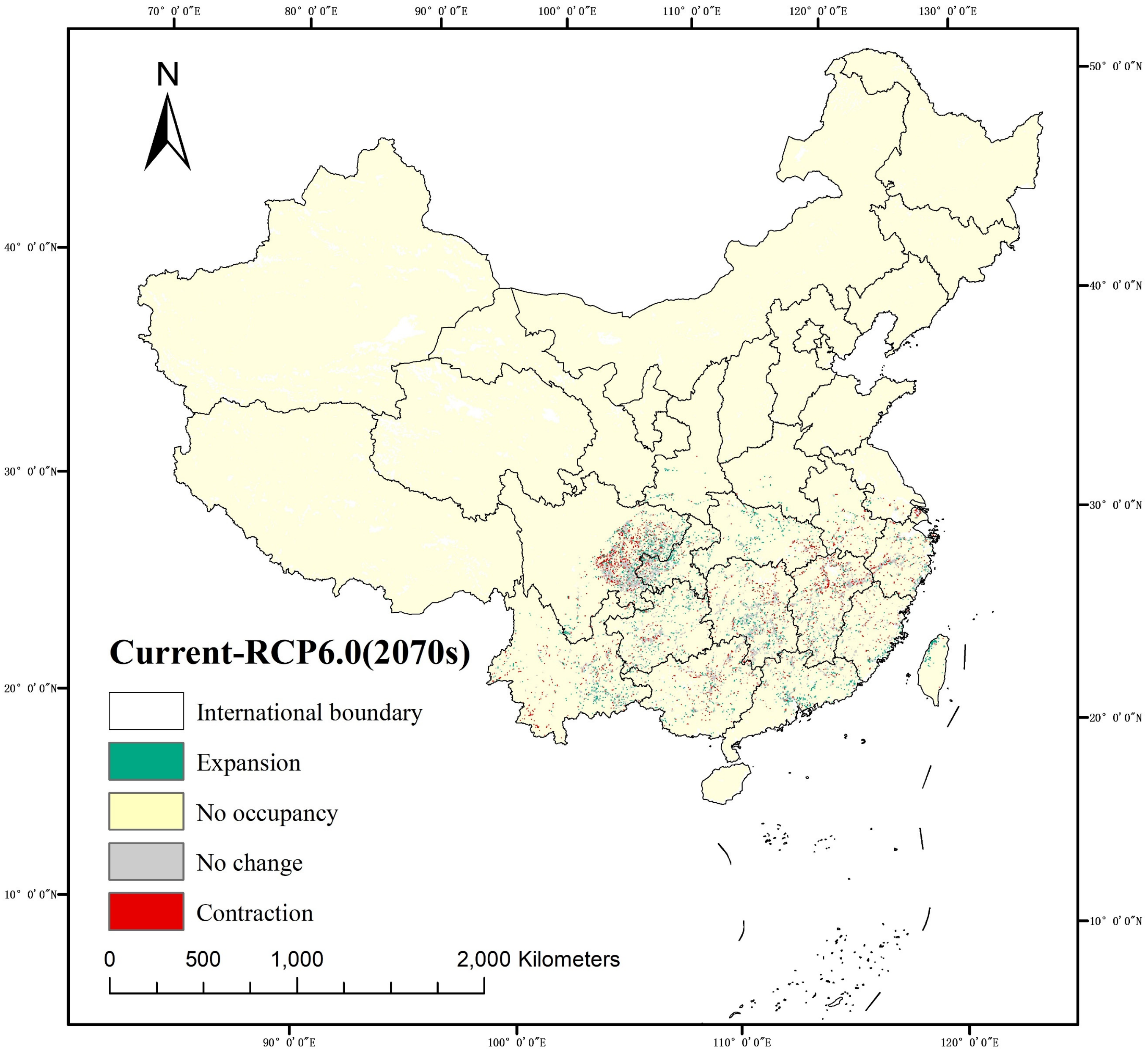
70° 0′ 0″E
80° 0′ 0″E
90° 0′ 0″E
100° 0′ 0″E
110° 0′ 0″E
120° 0′ 0″E
130° 0′ 0″E
50° 0′ 0″N
40° 0′ 0″N
30° 0′ 0″N
20° 0′ 0″N
10° 0′ 0″N
N
Current-RCP6.0(2070s)
International boundary
Expansion
No occupancy
No change
Contraction
0
500
1,000
2,000 Kilometers
90° 0′ 0″E
100° 0′ 0″E
110° 0′ 0″E
120° 0′ 0″E

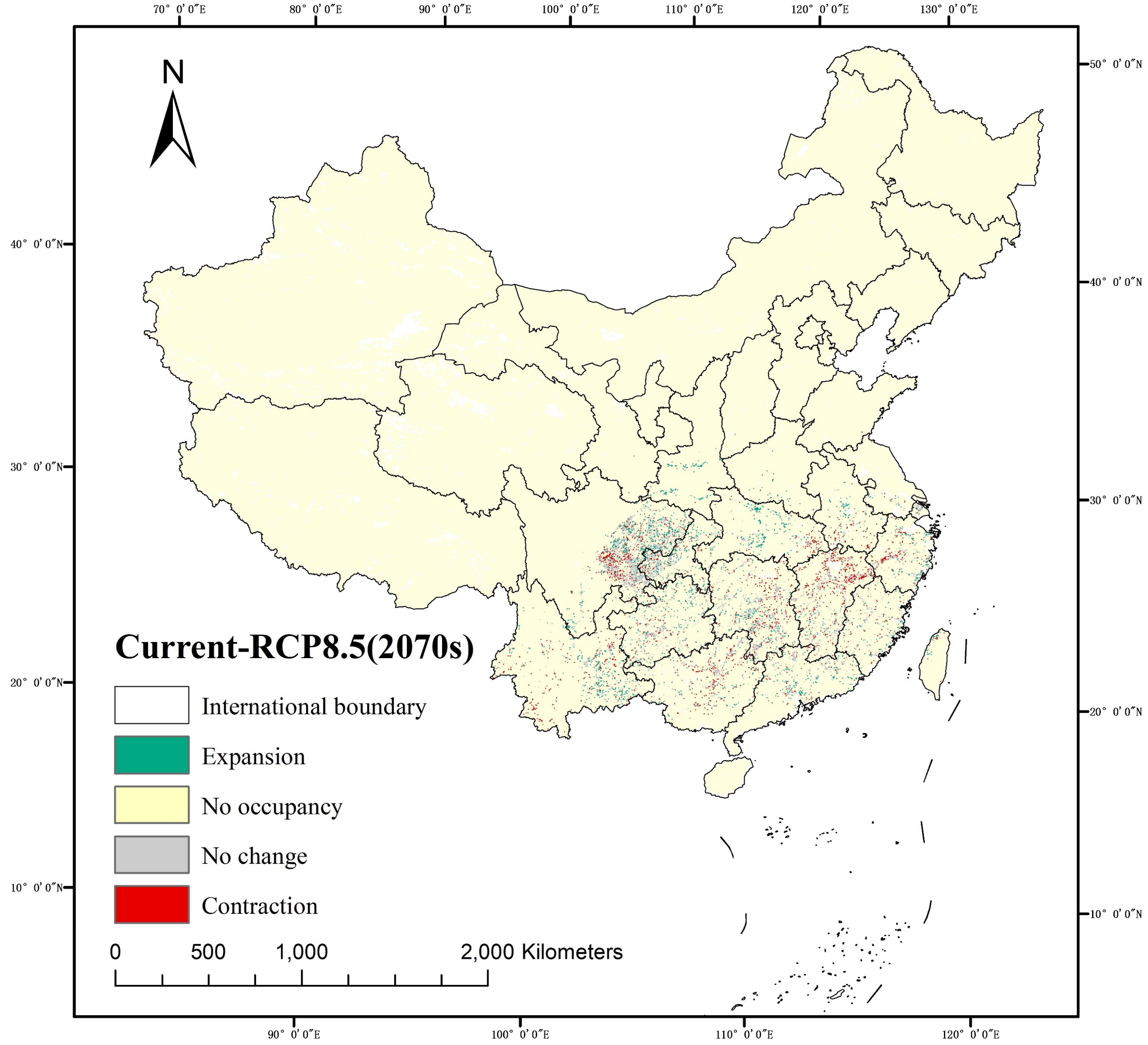

Figure S4. Changes in the distribution of *C.acuminata* under four scenarios from current to the 2050s and from current to the 2070s.
